# Robustness assessment of regressions using cluster analysis typologies: a bootstrap procedure with application in state sequence analysis

**DOI:** 10.1186/s12874-024-02435-8

**Published:** 2024-12-18

**Authors:** Leonard Roth, Matthias Studer, Emilie Zuercher, Isabelle Peytremann-Bridevaux

**Affiliations:** 1 Department of Epidemiology and Health Systems, Centre for Primary Care and Public Health (Unisanté), University of Lausanne, Route de La Corniche 10, 1010 Lausanne, Switzerland; 2 Centre LIVES, University of Geneva, Boulevard du Pont-d’Arve 40, 1205 Geneva, Switzerland

**Keywords:** Sequence Analysis, Cluster analysis, Regression analysis, Typology of trajectories, Association study, Statistical inference, Sampling variation, Sampling uncertainty, Bootstrap, Multilevel modelling

## Abstract

**Background:**

In standard Sequence Analysis, similar trajectories are clustered together to create a typology of trajectories, which is then often used to evaluate the association between sequence patterns and covariates inside regression models. The sampling uncertainty, which affects both the derivation of the typology and the associated regressions, is typically ignored in this analysis, an oversight that may lead to wrong statistical conclusions. We propose utilising sampling variation to derive new estimates that further inform on the association of interest.

**Methods:**

We introduce a novel procedure to assess the robustness of regression results obtained from the standard analysis. Bootstrap samples are drawn from the data, and for each bootstrap, a new typology replicating the original one is constructed, followed by the estimation of the corresponding regression models. The bootstrap estimates are then combined using a multilevel modelling framework that mimics a meta-analysis. The fitted values from this multilevel model allow to account for the sampling uncertainty in the inferential analysis. We illustrate the methodology by applying it to the study of healthcare utilisation trajectories in a Swiss cohort of diabetic patients.

**Results:**

The procedure provides robust estimates for an association of interest, along with 95% prediction intervals, representing the range of expected values if the clustering and associated regressions were performed on a new sample from the same underlying distribution. It also identifies central and borderline trajectories within each cluster. Regarding the illustrative application, while there was evidence of an association between regular lipid testing and subsequent healthcare utilisation patterns in the original analysis, this is not supported in the robustness assessment.

**Conclusions:**

Investigating the relationship between trajectory patterns and covariates is of interest in many situations. However, it is a challenging task with potential pitfalls. Our Robustness Assessment of Regression using Cluster Analysis Typologies (RARCAT) may assist in ensuring the robustness of such association studies. The method is applicable wherever clustering is combined with regression analysis, so its relevance goes beyond State Sequence Analysis.

**Supplementary Information:**

The online version contains supplementary material available at 10.1186/s12874-024-02435-8.

## Background

### Introduction

State Sequence Analysis, often referred to simply as Sequence Analysis (SA), is a set of statistical methods for studying longitudinal data [1]. It enables a holistic view of processes modelled as a succession of categorical states [2]. Over the past few years, SA has been increasingly used in health research for the study of trajectories, or pathways [3–5]. In these studies, the standard analysis identifies typical trajectories in two steps: by defining a dissimilarity measure to quantify the variation across trajectories [6] and by grouping similar trajectories with a cluster algorithm based on the pairwise dissimilarities [7]. Then, the relationships between covariates and the trajectories are studied by including the typology of trajectories into regressions, either as a dependent or independent variable [8]. This paper proposes to recognize the uncertainty involved in this standard framework and evaluate its impact on the analysis’ results.

Typologies are a popular tool as they summarize the information available by reducing the diversity of trajectories into a few ideal types (see Herle et al. [9] for a review of modelling strategies, including SA, to identify typical trajectories). For instance, this enables to highlight segments of the population that can be targeted for public health interventions [10]. While typologies are mainly descriptive by themselves, they also allow the inclusion of the complex concept of trajectories in subsequent inferential analyses. Such analyses consist for instance in investigating why individuals follow certain trajectories instead of others, or how previous trajectories influence later outcomes [11–13].

Assuming an ideal situation with no missing information, two major sources of uncertainty can impact the derivation and the subsequent use of a sequence typology. The first is linked to data reduction and the potentially imperfect assignment of sequences to clusters. The second is the sampling error, common to most statistical problems, as any sample is an inexact representation of the underlying population. The two issues are, however, closely interrelated, as we are less confident to make inference in a context with higher approximation risk.

These two sources of uncertainty might affect the analysis on two levels. First, the description of the processes observed in the data, which are generally inferred to the population. Second, it may lead to wrong conclusions when relating trajectories to covariates in subsequent regression models [7, 14–16].

As it will be established in the remaining of this section, previous methodological developments in SA have mostly focused on issues related to the data simplification induced by cluster analysis, and the associated assignment error. Issues related to the error in the estimation of the typology due to the sampling uncertainty have been on the other hand overlooked by the SA literature. However, this estimation error can have a serious impact on inference as it has been shown in the latent class literature [17–19]. In this article, we propose an innovative method to take sampling variation into account in the analysis. Our contribution goes beyond SA and is relevant for any studies combining cluster analysis with regressions.

### Data reduction risk

Before considering the impact of sampling error on sequence typology inference, we consider in more detail the uncertainty involved in building the typology. SA often aims to reduce the large diversity of sequences into a few ideal types, while losing as little information as possible. However, this data reduction might raise two kinds of issues that are again closely interrelated.

First, cluster analysis always produces a typology, and therefore imposes a structure on the data even if the data is not structured into subgroups [20, 21]. When there is a clear clustering structure in the data, i.e., when the observed sequences are grouped into clearly distinguished types, we expect the clustering procedure to recover, precisely enough, the underlying clustering structure. In this case, there should be little doubt about whether a given sequence belong to a given type or another. On the contrary, when the observed data is unstructured, i.e., when there is a lot of diversity across sequences with no homogeneity apparent anywhere, the grouping produced by the clustering, and the associated assignment of sequences to cluster, might be uncertain. In such context, small variations of the data could lead to very different typologies.

Second, once the typology has been created, one commonly assumes that all individuals in a given cluster are perfectly represented by their corresponding typical trajectory. As a result, all the remaining within-cluster variation is ignored. However, while some sequences might be close to their cluster centre, and therefore well-represented by the centre, others may be far away and poorly represented [7, 15]. Such data reduction is a strength, as many real-world problems can be simplified by uncovering fundamental structures, but it is also a risk. Indeed, one should not simplify the *relevant* variation of the trajectories. In such case, an *excessive* simplification could lead to a wrong description of the trajectories. Furthermore, it raises additional risks when the typology is used in subsequent analysis, such as regression models. Indeed, cluster analysis might ignore exactly the relevant variation to understand the relationships between trajectories and key covariates of interest. In such cases, the use of the typology in subsequent regressions might lead to wrong conclusions [7, 14–16].

Several tools have been developed to handle these two methodological issues. First, cluster quality indices are commonly used tools to validate a SA typology. They measure the quality of a partition, usually by relating the within-cluster homogeneity and/or the between-cluster separation [7]. They are useful to evaluate the statistical quality of a partition and allow comparing results from different clustering algorithms. However, these cluster quality indices lack clear interpretation thresholds [22, 23]. Furthermore, they only indirectly inform on the underlying clustering structure of the data, i.e., whether the data is organized in subgroups or not. Testing the obtained typology against clustering applied on non-clustered data generated by a null model provides such interpretation thresholds and allows a better inference on the quality of the observed typology [17].

Second, several authors have advocated for the use of other clustering approaches, including fuzzy clustering [24] and the “representativeness” approach [14]. In these methods, sequences may belong to more than one cluster, with gradual membership strength. It allows a better description of sequences that can be understood as a mixture of several ideal types. Those sequences are necessarily assigned to a single type with “traditional” crisp clustering such as Ward’s method or Partitioning Around Medoids. In addition, it might better capture the within-cluster variation [24]. The “representativeness” approach further allows describing complete outliers, i.e., sequences that are far from any types. According to the simulations presented in Helske et al. [14], these two approaches provide better estimates of the relationships between sequence typologies and dependent or independent variables, as they better account for the imperfect cluster assignment of individual sequences.

Finally, Unterlerchner et al. [16] proposed a procedure to measure the potential impact of the simplification conducted by cluster analysis on subsequent analyses. More precisely, the method measures the share of a statistical relationship between sequences and covariates without prior clustering, which is accounted for by the clustering. When the relationship is poorly accounted for by the clustering, one should be careful about the interpretation of the subsequent results.

Previous research has therefore focused on the issues stemming from the simplification of the data induced by the clustering itself. The proposals allow either measuring the extent of the simplification issue using cluster quality indices, or advocate for the use of other clustering approaches such as fuzzy clustering. However, none of these works aimed to address the issue of inference of the typology. The typology is ordinarily estimated using a sample of the population, and its generalization to the population is subject to error. In other words, the typology might be sample-dependent, meaning that a different sample could lead to a different typology.

### Sampling uncertainty

The proposals and developments introduced above emphasize that the typology should not be blindly relied upon, as the simplification may lead to wrong conclusions. These risks should be understood at two levels. First, one might draw wrong conclusions on the descriptive level by incorrectly summarizing the observed processes, because of excessive simplification or imprecise cluster assignment. Second, it might affect subsequent analyses making use of the typology.

However, these developments do not consider the sampling error. As in most statistical analysis, a given result may be specific to a given sample, and poorly represent the properties of the underlying population. One clear way to illustrate this—experienced by many researchers—is that if the sample is modified (by for instance removing corrupted data or adding new observations), chances are that the resulting typology will look somewhat different. Such changes in the typology may be explained by several reasons, which are closely related to the issues presented earlier. First, we can expect bigger changes in the typology when the data does not contain a clear clustering structure. Indeed, in such cases, several clustering solutions might describe the underlying structure equally well and the best solution could differ between samples resulting in different typologies. Second, when individual sequence assignment to clusters is dubious, this assignment is likely to change between samples. This is typically the case for sequences that can be seen as a mixture of ideal types. Third, and most obviously, a new sample will include new observations, which may lead to the estimation of a completely different typology. This is particularly expected when using small samples, as additional observations will have a stronger influence in this case.

This discussion highlights two points that are common to many statistical methods. The estimation process depends on the sample. In such context, we can expect more reliable estimation when the underlying structure is strong. Furthermore, we can also be more confident when using larger samples, as we have a more complete dataset. This is akin to the estimation of the population mean using the sample mean. In this case, the standard error depends on the variance (which can be linked to underlying clustering structure) and the sample size.

Sampling error raises further issues when the typology is used in subsequent regressions. All the methodological improvements previously mentioned still handle typologies as *measures,* i.e., singular realization of the partition of interest (even if their validity is then investigated). However, one can argue that they should be handled as *estimates*, i.e., random realizations of the partition of interest that can be reproduced to derive the distribution of instructive quantities. This recognizes the fact that exhaustive information is never available to derive the typology. One should therefore consider the estimation error of the typology in subsequent analysis to draw correct inference.

While not common in SA, several tools were proposed in the data mining literature. These propositions generally rely on the use of different kinds of resampling schemes to account for sample variation of the results. Monti [25] proposed a clustering procedure aiming to avoid sample dependence of the resulting typology, called consensus clustering. The method starts by creating several typologies of the same underlying population in many subsamples. Then, it looks for a consensus clustering between these typologies. While the method supports the creation of a robust typology, it does not allow considering the typology as estimate in subsequent regressions.

Other authors have proposed to measure the stability of the clustering across multiple subsamples of the data [26, 27]. Stability measures provide an estimate of the sample dependence of the results, and indirectly, of the underlying clustering strength of the data. For this reason, they are also used for cluster validation, as a complement to other validation techniques [28]. Generally speaking, these stability measures are estimated as follows [29]:

1. Cluster the original data to obtain the typology to be evaluated.
2. Resample or alter the original data.
3. Cluster the new sample using the same clustering method as in step 1.
4. Repeat steps 2 and 3 many times.
5. Measure the variations of the clusterings between the data resamples.

Several propositions were made, which differ according to the resampling procedure (bootstrap, data jittering, etc.), and the measurement of the variation of the clustering (see Liu et al. [30] for a review). In this article, we rely on the method proposed by Hennig [26] called “Cluster-wise stability assessment”. This method primarily uses random sampling with replacement from the data, i.e., bootstrapping, to replicate the original sample as no true underlying distribution is known. The strength of the approach is to estimate the stability separately for each type. Indeed, while some types might be very well-defined in the data, others may be more dubious. This is achieved by measuring the number of times a given type was recovered in the bootstraps using the Jaccard coefficient [31]. A type is considered as recovered when the same observations were regularly clustered together in the bootstrap clusterings.

The aim of this paper is to extend this approach to the use of the typology in the subsequent step of traditional SA, i.e., to assess the robustness of the relationship between sequence patterns and covariates. This assessment delivers new model diagnostics and alternative estimates for an association of interest. We focus on the situation where the clustering is used as dependent variable in a regression.

The content of the article is outlined as follows. We begin by presenting an illustrative application, which serves as case study throughout this paper. A classical SA framework used to construct a typology of trajectories on this illustration data and estimate its association with covariates is then described, implemented, and commented. In the Methods section, we lay out a novel approach to assess the robustness of typology-based inference studies in two steps. First, a bootstrap procedure to reproduce the typology over an ensemble of perturbed datasets. This step is common with previous works on the evaluation of cluster-wise stability. Second, a complementary multilevel model to pool estimates obtained from the typology replications. In the Results section, we show how implementing this methodology allows to revisit the illustrative application and to derive new quantities, which inform on the impact of sampling uncertainty on the original results. We also consider a range of possible relationships between clusters and covariates to further clarify the significance of the methods. Finally, we demonstrate how our contribution permits to gauge the analysis’ reproducibility and contextualise it inside a general cluster validation framework.

## Illustrative application

### Problem setting

The presentation of the methodological developments is illustrated by applying them to the study of diabetic patients’ healthcare utilisation trajectories. Diabetes is one of the most common chronic diseases of our times, with an estimated global prevalence of 10.5% among 20–79 years old persons in 2021 [32]. It is considered an ambulatory care sensitive condition (ACSC), meaning that adverse and costly events such as emergencies and hospitalisations for diabetes complications can be avoided by high-quality primary care [33–35]. We are interested in studying how compliance with recommended screening processes is related to subsequent healthcare utilisation patterns. For illustration purposes, we concentrate our investigation on regular lipid screening.

Studying healthcare utilisation patterns as longitudinal processes provides a holistic perspective allowing to situate each healthcare event within the whole trajectory [13]. This is key as, for instance, a hospitalisation should be interpreted differently if it occurs repeatedly over time. Moreover, healthcare utilisation trajectories can in some cases be modelled as a succession of categorical states and hence, studied with SA methods. To the best of our knowledge, two previous studies have applied SA in the context of diabetes and health services research, with the aim of identifying typical care pathways and characterizing the patients on each pathway [12, 36].

### Study design, population and measurements

We used data from a prospective cohort study (CoDiab-VD) of non-institutionalised adult patients with diabetes diagnosed for at least a year and residing in the canton of Vaud, Switzerland [37]. We considered the individuals recruited by community-based pharmacies in 2011–2012 and followed-up yearly until 2017. This cohort included 519 participants at baseline, out of which 428 participated to at least one follow-up. We focused on the 348 participants with no more than two missing observations during the follow-up period. The data were collected by postal questionnaires encompassing different aspects of participants’ health status, diabetes care and daily life.

The trajectories of healthcare utilisation were measured between 2013 and 2017 by looking at self-reported emergency visits, hospitalisations, and deaths (retrieved from registries), resulting in the following states: *no utilisation*, *emergency visit*, *hospitalisation*, *emergency visit & hospitalisation*, *dead*, and *missing*. As multiple occurrences of emergency visits and hospitalisations during a year were relatively rare (11% and 7% of those cases, respectively), we grouped them together with single events, thus limiting the number of distinct states.

Quality of diabetes care was measured by looking at compliance with processes of care, which should be conducted yearly [38]. We looked at six processes: foot examination, microalbuminuria screening (from urine sample), lipid testing (from blood sample), influenza immunisation, eye examination (by an ophthalmologist), and glycated haemoglobin (HbA1c) measurement. For each of these processes individually, it was considered that the patient complied to the recommendations if the process was reported for two successive years, i.e., both at baseline in 2011 and in the first follow-up in 2012.

In our analysis, we controlled for a set of confounding factors based on existing literature, the investigators’ domain-specific knowledge, preliminary analyses (not reported here) and statistical considerations [39]. The selected variables, detailed in Table S1 in the Supplementary Material, were all collected before the start of the trajectories: age category, household income, diabetes treatment, diabetes-related complications, and comorbidities. Covariates such as gender and education were discarded as they were associated neither with the outcome nor with the exposure in this context.

### The typology

**Fig. 1:**
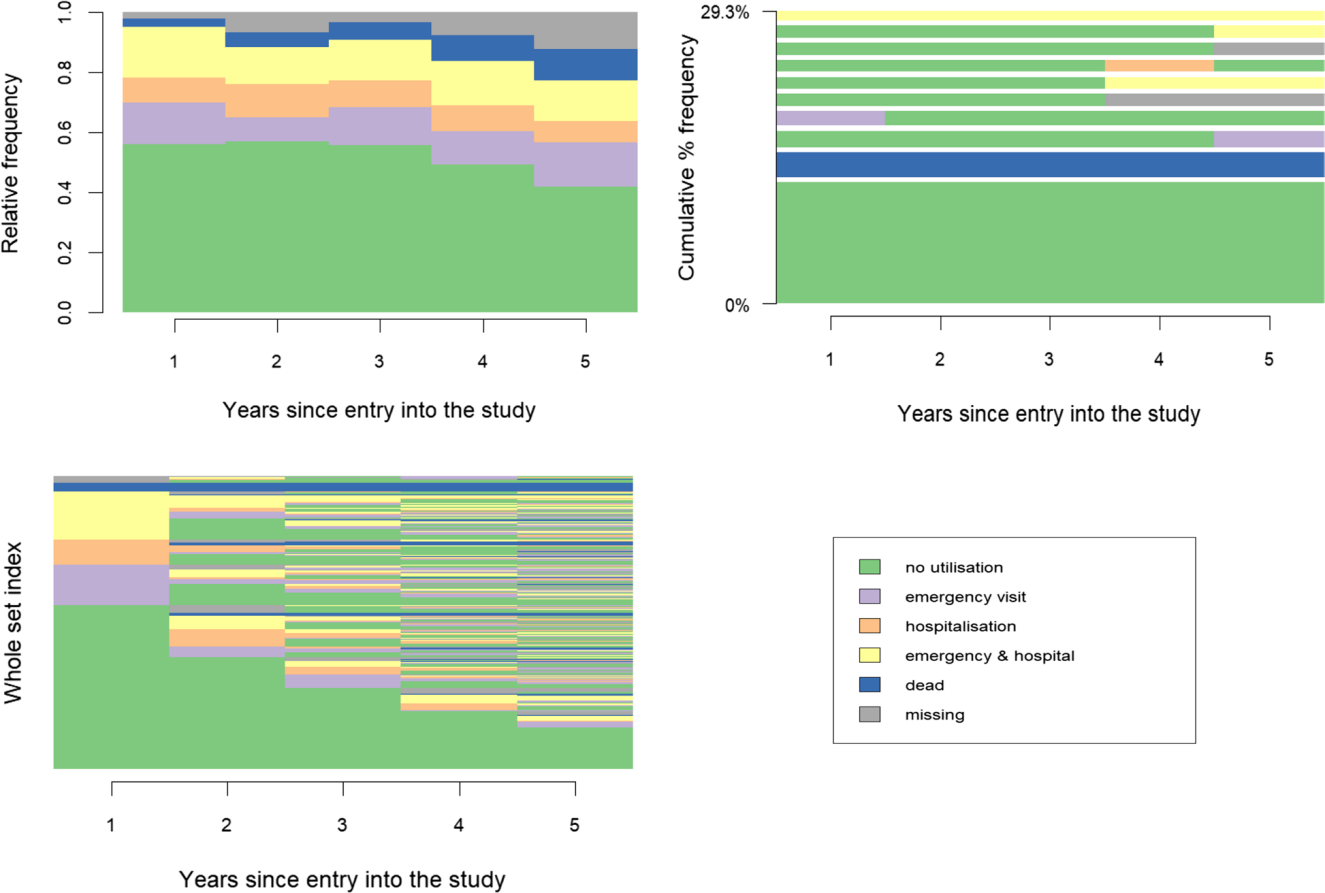
Exploratory sequence analysis for the 348 trajectories of healthcare utilisation. X-axis represents the five years of follow-up (2013 – 2017). Top left is the state distribution over time. Top right are the 10 most frequent sequences and how often they occurred. Bottom left are all individual sequences ordered

Before presenting our methodological proposal, let us follow a standard SA framework to derive a typology of healthcare utilisation trajectories. Figure 1 presents the individual sequences that serve as basis for our illustrative application, together with their state distribution, which shows the cumulative proportion of participants in each state at a given time point. The most frequent trajectories correspond to patients reporting no emergency visits nor hospitalisations throughout follow-up and patients who died soon after inclusion in the cohort.

We used Optimal Matching as the dissimilarity measure, which focuses on duration in each state and their ordering [6]. As transitions from no utilisation to emergency visit or hospitalisation were more common than to death directly, we used substitution costs based on the observed transition rates and set indel costs to half the maximum substitution costs [40]. Following Halpin [41], we used a maximal substitution cost between missing states and any other states, including other nonresponses, to avoid considering missing data as a factor of similarity between trajectories. Clustering was performed with Partitioning Around Medoids [42]. We selected the solution in three groups by looking at the best quality of partitioning according to the average silhouette width and the Calinski-Harabasz index (CHI) [7].

**Fig. 2:**
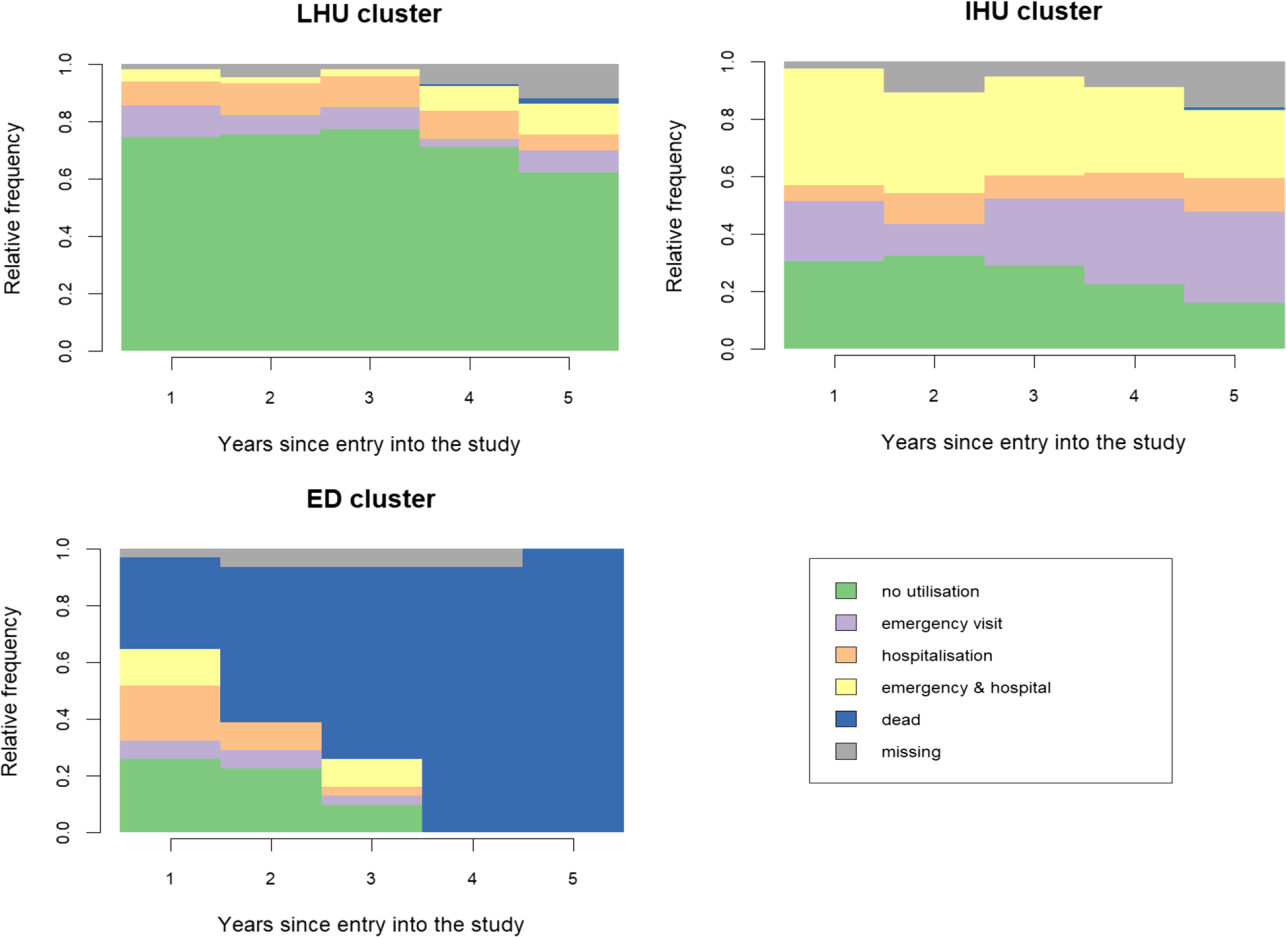
State distribution plots for the three clusters identified. X-axis represents the five years of follow-up (2013—2017). Top left is the low (intermittent) healthcare utilisation cluster (*n* = 206). Top right is the high (intensive) healthcare utilisation cluster (*n* = 111). Bottom left is the early death cluster (*n* = 31)

Figure 2 presents the resulting typology using state distribution plots. The clusters identified correspond to individuals with “low” (*n* = 206; LHU cluster) and “intensive” (*n* = 111; IHU cluster) healthcare utilisation, as well as those who died early in the study (*n* = 31; ED cluster). The largest cluster contains trajectories congruent with a diabetes under control, and the two others feature high levels of adverse healthcare events. Figure S2 in the Appendix shows the most representative sequences in each cluster.

### Association study

Table 1 describes the variables from our illustrative application, for the whole sample and by cluster. We picked as case study the association between compliance with lipid screening recommendations and subsequent healthcare utilisation patterns, controlling for known confounders. However, the same reasoning and methods could be applied to any covariate.

**Table 1:** Demographic, socioeconomic, health characteristics and processes of care indicators of study participants, by cluster

		**Whole sample**	**LHU**	**IHU**	**ED**	***p*-value**
**Total**	N	348	206	111	31	
	%	100	59.2	31.9	8.9	
Age* (years)	<65	48.9%	55.3%	45.9%	16.1%	<0.001
	65–74	37.1%	35.4%	38.7%	41.9%	
	>=75	14.1%	9.2%	15.3%	41.9%	
**Sex**	Female	36.8%	38.3%	37.8%	22.6%	0.228
	Male	63.2%	61.7%	62.2%	77.4%	
**Educational level**	Basic	14.9%	15%	16.2%	9.7%	0.963
	Secondary	57.5%	56.8%	57.7%	61.3%	
	Higher	25.9%	26.7%	24.3%	25.8%	
	Other	1.7%	1.5%	1.8%	3.2%	
Household income*	Low	17.5%	15%	16.2%	38.7%	0.042
	Lower-middle	26.1%	26.7%	27.9%	16.1%	
	Upper-middle	28.4%	30.1%	27%	22.6%	
	High	17%	19.4%	15.3%	6.5%	
	Unknown	10.9%	8.7%	13.5%	16.1%	
Diabetes treatment*	OAD	52%	56.3%	47.7%	38.7%	0.186
	Insulin	20.4%	18.4%	20.7%	32.3%	
	Both	26.7%	24.3%	31.5%	25.8%	
	missing	0.9%	1%	0%	3.2%	
Diabetes-related complications* (N)	Mean	0.7	0.6	0.8	1	0.014
	SD	0.9	0.9	1.1	0.8	
Comorbidities* (N)	Mean	1.9	1.7	2.1	2.3	0.003
	SD	1.3	1.2	1.5	1.4	
**Foot examination**	Yes	56.9%	54.4%	61.3%	58.1%	0.493
	No	43.1%	45.6%	38.7%	41.9%	
**Microalbuminuria screening**	Yes	62.9%	66%	60.4%	51.6%	0.571
	No	29%	26.7%	30.6%	38.7%	
	Unknown	8%	7.3%	9%	9.7%	
Lipid testing*	Yes	92.5%	95.6%	88.2%	87.1%	0.022
	No	6.6%	3.4%	10.8%	12.9%	
	missing	0.9%	1%	0.9%	0%	
**Influenza immunisation**	Yes	60.6%	58.3%	64%	64.5%	0.482
	No	39.1%	41.7%	36%	32.3%	
	missing	0.3%	0%	0%	3.2%	
**Eye examination**	Yes	89.4%	87.4%	91.9%	93.5%	0.225
	No	10.1%	12.1%	8.1%	3.3%	
	missing	0.6%	0.5%	0%	3.3%	
**HbA1c measurement**	Yes	82.2%	84%	80.2%	77.4%	0.542
	No	4.3%	3.4%	4.5%	9.7%	
	Unknown	13.5%	12.6%	15.3%	12.9%	

*Legend:* Bivariate relationships are evaluated with chi-squared tests for categorical variables and *ANOVA* for numerical variables. *LHU* cluster is low healthcare utilisation, *IHU* is intensive healthcare utilisation, *ED* is early death. *OAD* stands for oral antidiabetic medication. A star (*) indicates that the variable is selected in the regression models

To investigate such relationship, one generally estimates regression models specifying the typology (through cluster membership) as the dependent variable. This can be achieved either using multinomial regression or a set of logistic regressions. We rely on the second approach, where a separate logistic regression is used to estimate the probability of belonging to each cluster versus any other. By doing so, coefficients for a specific cluster do not directly depend on the coefficients estimated for the other clusters, which will be key for assessing their robustness thereafter. Indeed, our proposed method focuses on characterising the data at the cluster level, and thus, emphasizing the attributes of individuals in that specific cluster compared to the rest of the sample.^1^

**Fig. 3:**
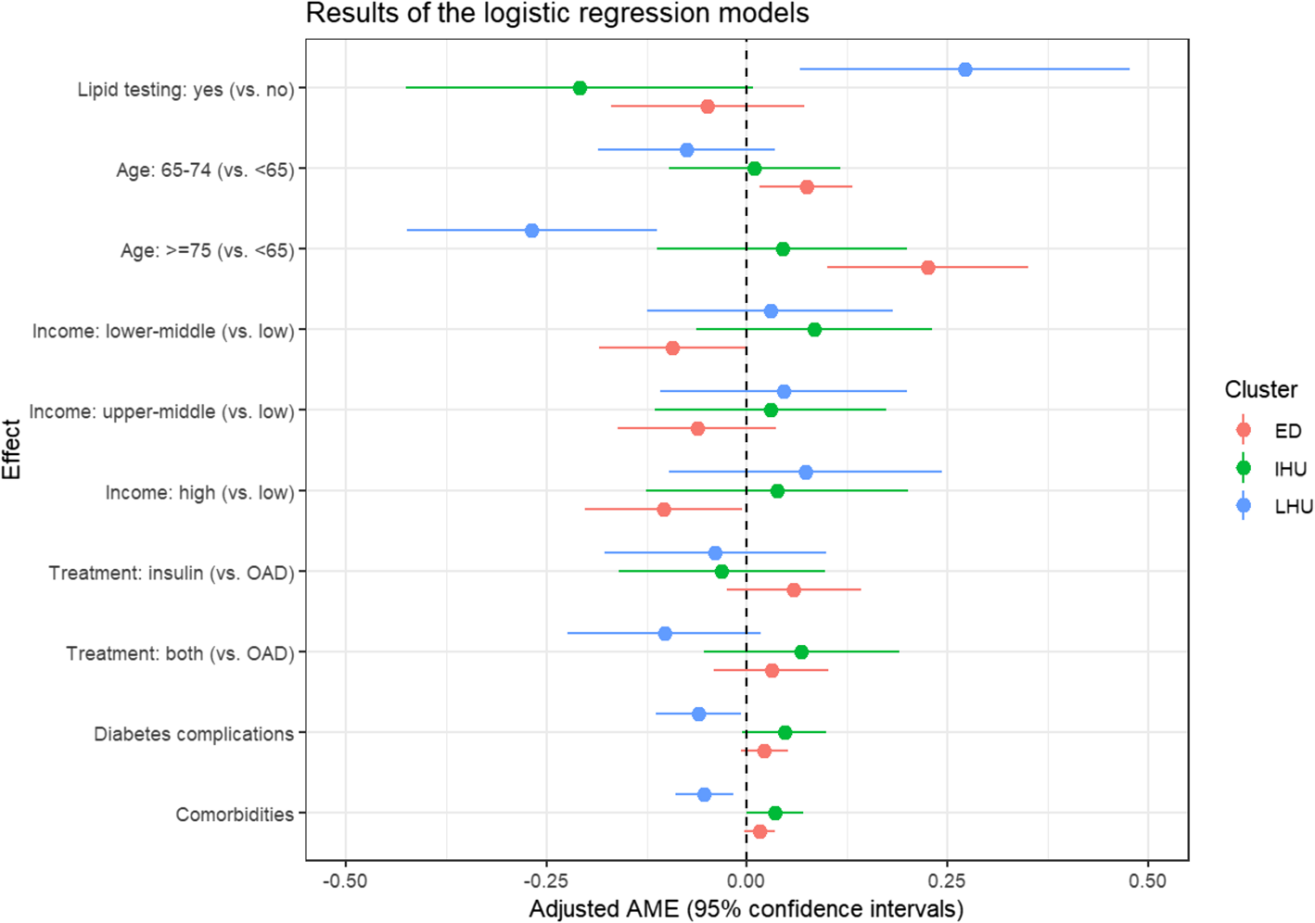
Results (*n* = 342) from the three logistic regression models with membership to a specific cluster as the dependent variable. LHU stands for low healthcare utilisation, IHU for intensive healthcare utilisation and ED for early death

Figure 3 presents the average marginal effects (AMEs) of the multivariable logistic regressions, which measure the expected change in probability of belonging to the trajectory group for a change in the level of a variable. The numerical estimates are provided in Supplementary Table S3. There were less than 2% missing values (*n* = 6) in the regression models overall, so the corresponding individuals were ignored. We rely on AMEs here for two reasons. First, they can be interpreted on the probability scale, and are therefore easier to comprehend. Second, they can be compared across subsamples and studies, which is not the case for the logistic regressions’ coefficients or odds ratios [43].

To avoid overloading the illustration, we focus on the probability to be in the “low” healthcare utilisation trajectory group against any other. Any other combination of clusters is possible, as demonstrated with further analyses, and the procedure is alike. Thus, our main quantity of interest is 0.271 (95% CI 0.066 to 0.476), which represents the expected change in probability of reporting low healthcare utilisation during follow-up for diabetic patients who complied with lipid screening recommendations, controlling for known confounders. These values indicate evidence towards a positive effect of regular lipid testing on subsequent healthcare utilisation patterns.

However, this inference does not account for the sampling error, which could impact the results’ reliability. Indeed, as introduced earlier, the typology is estimated based on the available sample, and the uncertainty involved in the estimation has carry-over effects on the subsequent use of the typology in a regression model, especially if the sample is small as is the case here. Next, we detail how resampling from the data allows to assess the robustness of the regression results and to derive new quantities that more adequately account for the sampling variation and its impact on a standard SA framework.

## Methods

### Bootstrap replicates of the typology

Building on the framework introduced by Hennig [26], the proposed method relies on non-parametric bootstrapping to account for the degree of uncertainty involved in the clustering procedure and the associated regression models. The bootstrap samples are used to derive new partitions that can be seen as independent and identically distributed random variables in the space of all partitions, with respect to the original sample and the cluster algorithm [44]. Then, new quantities are estimated based on these replications of the typology, thus allowing to assess the robustness of the original regressions.

We call our proposed method the Robustness Assessment of Regressions using Cluster Analysis Typologies (RARCAT). Specifically, this procedure works as follows:

1. A random sample with replacement is drawn from the data.
2. The bootstrap sample is clustered applying the exact same clustering procedure as the one used in the original analysis, which implies using the same distance measure, cluster algorithm, and method to determine the number of clusters.
3. A separate logistic regression predicting membership probability in each group is estimated.
4. The AME of each covariate on the probability to be assigned to a given type is retrieved for all sequences belonging to this type.
5. These steps are repeated *N* times, with *N* typically large.
6. The individual AMEs from step 4 are pooled using a multilevel modelling framework.

As is customary, the bootstrap samples drawn in step 1 have the same size as the original sample. Individuals can appear more than once in a bootstrap sample, and the average number of distinct observations is approximately 63.2% [45].

Hennig [26] proposed to use the new partitions derived in step 2 to evaluate cluster-wise stability by measuring the quality of preservation of clustering solutions across perturbed datasets through average Jaccard similarities. We go one step further and use the bootstrap partitions to estimate each time new regression models replicating the original ones. The form of the regression equations stays identical across bootstraps, with the same controlling factors (if applicable) and only the cluster membership variable changing depending on the new partitions.

**Fig. 4:**
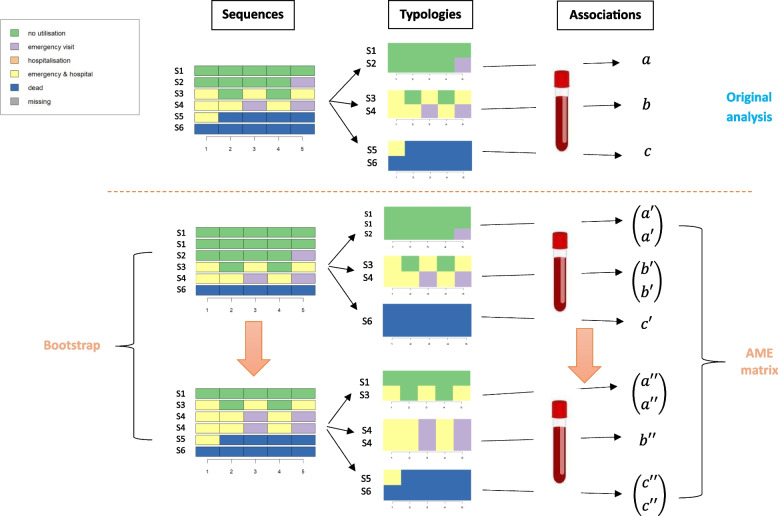
Diagram illustrating the proposed bootstrap procedure

Figure 4 outlines the first steps of RARCAT with a toy example of six trajectories (S1 to S6, the x-axis representing the time alike the years in Fig. 1 for instance). The upper part – above the dotted line – portrays the original analysis. The six sequences are clustered in three groups, which mirror the three trajectory groups from our illustrative application. The letters *a, b* and* c* denote the results from three logistic regressions to estimate the relationship between a covariate of interest (depicted as blood screening) and the typology. The lower part portrays two bootstrap replications of the analysis. In both bootstrap samples, one sequence is not drawn, and one sequence is drawn twice. This translates into two new typologies.

The typology in each bootstrap sample should be derived with the same procedure as the one originally applied. This implies using the same distance matrix, cluster algorithm, and the same method to determine the number of groups *K* [26]. The number of groups *K* deserves a special attention. If we consider that this is a fixed property, defined a priori or theoretically set, we can use the same *K* value in each bootstrap. However, if we consider that *K* is *estimated* from the data, for instance by maximizing a cluster quality index such as the CHI, then we should further consider the estimation error, and the associated sampling variation, stemming from the estimation of the number of groups. For sake of simplicity, we report here the results when fixing *K* to the value from the original analysis, i.e., *K* = *3*. However, we also report the results when estimating the number of groups by maximizing the CHI in each bootstrap as an alternative specification of RARCAT.

Crucially, while the first bootstrap typology in Fig. 4 is very close to the original typology, the second one is rather dissimilar. Indeed, a trajectory with multiple hospitalisations and emergency visits (S3) is now clustered together with the no utilisation trajectory. This illustrates a critical aspect of the method described in this section. The resamples can lead to new partitions that are not directly comparable across bootstraps. To circumvent this issue, the trick is to consider the regression results at the individual level in the bootstrap. This approach resembles the one used by Hennig [26], where points or sequences are compared in a pairwise manner between bootstrap replicates and the clustering to be evaluated. We rely on the same strategy and assign the regression estimate—such as the AME from our illustrative application—to every individual in a cluster. This is demonstrated in Fig. 4, where each bootstrap regression is linked to one or two estimates, depending on the number of distinct trajectories in the corresponding cluster. While these estimates are the same inside a regression model, assigning them to individuals allows to examine the stability of the results across bootstraps at the individual level. Thus, we can compare the original regression estimates to the bootstrap ones without worrying about cluster membership in the new samples, by centring the analysis on the original typology. This is called for because there is no trivial way to map clusterings across bootstraps.

To further illustrate this, let us consider the case of the two sequences S3 and S4 originally belonging to the middle cluster in Fig. 4. Their *b*, which represents the relevant regression estimate, will be assessed with the quantities *b’* from the first bootstrap, which are unlikely to differ much due to the relative preservation of the clustering, and thus of the associated regression models, but also with the quantities *a’’* and *b’’* from the second bootstrap, which may well differ markedly. Similarly, the robustness of *a* is assessed using the quantities *a’* and *a’’*, with the former being likely closer to the original value than the latter. Finally, *c* will be assessed with the effects *c’* and *c’’*, which should both stay close to the original value as the corresponding clusters are little impacted by the resampling.

In Fig. 4, five individual quantities are obtained for each bootstrap sample, as one sequence was missing from the sample each time. In practice, the regression results retrieved are the estimated AMEs and their standard errors (SEs). We focus on the AMEs and not the individual marginal effects to reflect the fact that the inference is done at the cluster level in the analysis. The AMEs can be interpreted here as the tendency for a sequence to be clustered with trajectories characterized by high (for positive AMEs) or low (for negative AMEs) rates of blood screening.

Thus, the output of this procedure for any association with a covariate of interest is an AME matrix (and its SE equivalent) of dimension *M x N*, where *M* is the number of individuals in the original sample and *N*, the number of bootstrap replicates. If a given individual is sampled in a given bootstrap, the entry in the AME matrix corresponds to the expected change in the probability of belonging to a certain cluster in this bootstrap for a change in the level of the covariate. Otherwise, if an individual is not sampled in a bootstrap, the corresponding entry is empty.

When the values estimated in a bootstrap are close to the ones obtained from the original regression model, it implies that the corresponding clusters’ characteristics are similar. This can for instance be expected for the first bootstrap replication in Fig. 4, but not for the second. Furthermore, individuals who are in-between types are more likely to have their AMEs oscillate from one bootstrap to the other based on which cluster they are assigned to. Thus, the estimated AMEs might vary across bootstraps depending on the influence that the sampling variation has on the association of interest. In extreme cases, the estimated value can even be of opposite sign compared to the original cluster.

Next, we propose a complementary multilevel model to aggregate and summarize the information obtained from the bootstrap procedure.

### Pooling effect sizes

One way to think about the bootstrap replications is as new studies that attempt to estimate the same effects each time. Thus, our objective is akin to a meta-analysis. In meta-analyses, quantitative information from related studies is synthetized by combining the study estimates of a particular effect of interest and producing a summary estimate of effect [46]. Statistical methods commonly used for meta-analysis are multilevel models [47, 48].

It is appropriate to apply these methods in our situation as there is an inherent nested structure in the results from the bootstrap procedure. Indeed, the estimated quantities are dependent on the bootstrap sample and on the individual. This can be related to a special case of meta-analysis, where primary studies are reporting multiple effect sizes—one per country for instance [49]. Here, multiple random effects are necessary to account for the dependency structure. Crucially, these random effects are not nested within one other, but rather crossed. Thus, a strict hierarchical model is not proper, and we implement instead a cross-classified random effects model [49]. In our setting, the crossed random factors signify that each effect size belongs to two dependency structures conjointly, the bootstrap and the individual, as is indicated in the model described hereafter.

This model is used to assess the robustness of an association between cluster membership and a covariate of interest. In our illustrative application, the cluster would be the low healthcare utilisation trajectory group and the covariate, regular lipid testing. Thus, we extract from the output of the bootstrap procedure an AME matrix of dimension *m x N* corresponding to a cluster with *m* < *M* individuals in the original typology.

Let *Y*_*ij*_ be the estimated AME for individual *i є {1, …, m}* in the reference cluster and bootstrap replicate *j є {1, …, N}* of the sample. The level 1 and level 2 equations are:

Level 1:

1 $${Y}_{ij}={a}_{ij}+{e}_{ij}$$

Level 2:

2 $${a}_{ij}={a}_{00}+{u}_{i}+{v}_{j}$$

- *a*_*ij*_ is the intercept for individual *i* and bootstrap sample *j*.
- *a*_*00*_ is the overall intercept (the pooled AME).
- *u*_*i*_ is the random effect representing the deviation from the overall intercept of individual *i*.
- *v*_*j*_ is the random effect representing the deviation from the overall intercept of bootstrap sample *j*.
- *e*_*ij*_ is the residual error proportional to the standard error of *Y*_*ij*_.

Additionally, we assume that:

- *e*_*ij*_ ~ *N (0, σ*^*2*^_*e*_*)*
- *u*_*i*_ ~ *N (0, σ*^*2*^_*u*_*)*
- *v*_*j*_ ~ *N (0, σ*^*2*^_*v*_*)*

Lastly, all random terms are assumed to be independent. To derive the quantities of interest in these equations, a linear mixed-effects model using restricted maximum likelihood is fitted [50]. As it is common for meta-analysis, the effect sizes in this model are weighted depending on their SEs, which were saved during the bootstrap procedure [48]. These weights are specified as inverse-variance weights, such that the more uncertainty in an effect size, the less weight it gets.

Different parameters are estimated with this model. First, the overall intercept fixed effect *a*_*00*_, which represents the pooled AME. This parameter is the mean change in cluster membership probability for a change in the level of the covariate of interest over all bootstraps and all individuals belonging to the reference cluster in the original typology. It is estimated together with its SE, which diminishes asymptotically as the number of bootstrap samples *N* increases. Indeed, in this context, the SE measures the expected error of estimation related to the finite number of bootstraps. Previous studies have recommended up to *500* bootstrap replications to attain a satisfactory precision around the estimates (e.g., [51]). This also applies in our case, and we run RARCAT with *N* = *1′000* in this paper to be on the conservative side. Whether this number is large enough to ensure that a new procedure with the same settings will output similar values is checked in the results section.

Second, the variance components of the two random effects. The variance component of the bootstrap random effect *v*_*j*_ is particularly important as it directly informs on the sampling variation and its impact on the regression results. It allows to construct a prediction interval (PI) for the value of the parameter of interest in a new sample [52]:

3 $${\widehat{a}}_{00}\mp {t}_{N-2}\sqrt{{{s}_{v}^{2}+SE\left({\widehat{a}}_{00}\right)}^{2}}$$

where:

- *â*_*00*_ is the estimated value of the pooled AME *a*_*00*_
- *SE(â*_*00*_*)* is the standard error of *â*_*00*_
- *s*_*v*_ is the estimate of the between-bootstrap standard deviation *σ*_*v*_.
- *â*_*00*_ is the estimated value of the pooled AME *a*_*00*_
- *t*_*N-2*_ is the 100(1—$\frac{\beta }{2}$) percentile of the *t* distribution with *N-2* degrees of freedom, where *N* is the number of bootstrap samples and $\beta$ is usually chosen as 0.05, to give a 5% significance level and thus, a 95% PI.

This prediction interval informs on the variation across bootstraps of the estimates related to the clustering and associated regression models, and thus, allows to assess the analysis’ results.

Concerning the individual-specific random effects *u*_*i*_, a large deviation from the overall intercept means that the AMEs estimated in the bootstrap procedure for a given individual were often divergent from AMEs for the other individuals assigned to the same cluster originally. It is a strong indication that this individual has an outlier trajectory compared to the cluster central trajectories, i.e., either an atypical trajectory or a trajectory in-between types. Such individual sequences were not robustly assigned to a cluster, which perturbs the estimation of their AMEs across bootstraps. On the other hand, a small variance for the individual-specific random effects indicates a homogeneous cluster. Assuming individual-specific random effects means that the true effect differs from individual to individual, with the pooled AME corresponding to an average individual in the cluster. Identifying individuals diverging from their cluster centre is typically of interest and can be achieved by looking at the estimated individual random effects. It allows to further investigate and understand the limits of the statistical analysis. We highlight this in our results section.

The proposed multilevel model relies on assumptions that are not necessarily met. Previous work indicated that the results are generally robust to moderate violations of these assumptions, as should be the case here [53]. We verify this by testing two alternative specifications of the model. First, the model assumes a normal distribution of the individual random effects. While modelling the variability across individuals as random around a population average seems sensible in our situation [54], another option could be to avoid modelling it directly by using individual-specific fixed effects, which implies a common effect for each individual—with the variation in the estimates only due to chance [55]. Thus, the focus of the estimation becomes the specific individual parameters, whilst the variability across bootstraps remains random.

Second, in some extreme cases such as very strong associations, the assumption of normality of the level 1 residuals might not be proper because the expected change in probability would be close to one or minus one. A solution is to transform the AMEs with an inverse hyperbolic tangent function in Eq. (1), to make them unbounded [56]. The relevant quantities are then obtained with the inverse transform, and their SE approximated with the Delta method. Such a model, identical to the main one apart from the transformation, is also tested as an alternative specification.

This whole assessment procedure was applied to our illustrative application. Besides the main association of interest, we also investigated the values obtained with different combinations of clusters and independent variables. This was done as further investigations to get a better sense of the magnitude of the multilevel model parameters. It also showed that RARCAT is easily reproducible with different data applications.

The proposed method relies on bootstrap and might therefore suffer from the usual limitations of resampling methods, including in the situation of low sample size or in the presence of outliers. In these cases, standard bootstrap diagnostic tools can be used to document the potential impact on the results [57]. An example of such procedure for outlier detection is proposed as Supplementary Material.

Finally, RARCAT’s effectiveness may be evaluated through a training/testing approach. Indeed, the bootstrap procedure simulates how the model would behave on new, unseen data, so the expectation is that the quantities obtained from it are more robust than the original ones in respect to out-of-sample validation. As this approach is treated as a potential confirmation of the one presented in this paper, the corresponding method and results are detailed as Supplementary Material.

All computational and statistical analyses were performed using the software *R* v4.3.1, with the help of packages *TraMineR*, *WeightedCluster*, *margins, boot, fpc* and *lme4* among others. RARCAT will be implemented and distributed into popular SA packages and publicly released once our work is published.

## Results

Starting from the typology identified in our illustrative application (Fig. 2), we used 1′000 bootstraps to evaluate the cluster-wise stability. Estimated Jaccard coefficients were 0.95 for the low utilisation cluster, 0.92 for the intensive utilisation cluster and 0.96 for the early deaths cluster. These values indicate high cluster-wise stability, meaning that most of the individuals belonging to any of the three clusters in the original partition tend to be clustered together again in the bootstrap partitions.

**Fig. 5:**
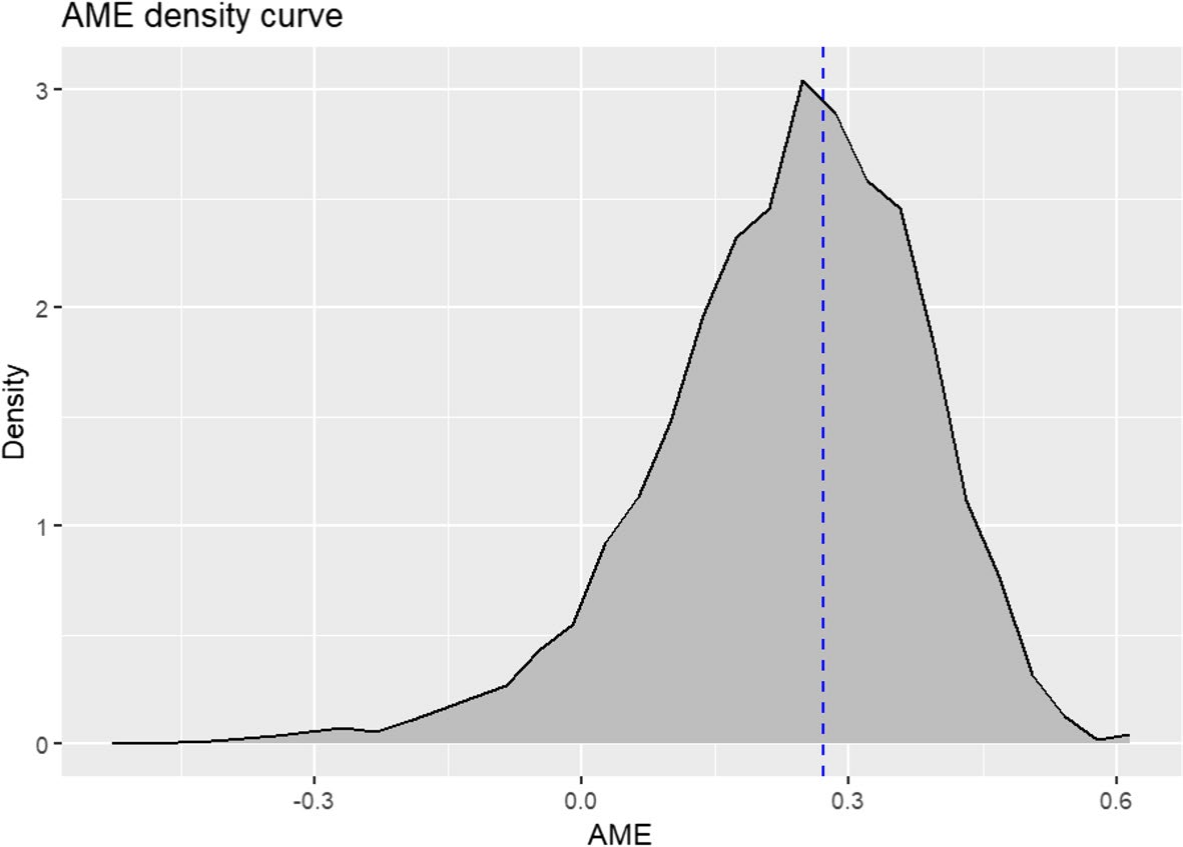
Density plot for 129′829 AMEs recovered from a 1′000 bootstrap procedure. The AMEs are for the association between regular lipid screening and cluster membership for patients belonging to the low healthcare utilisation trajectory group in the reference typology (*n* = 206). The dotted blue line corresponds to the estimated effect in the original analysis

The 1′000 bootstrap replicates of the typology were also used to estimate the relationships between healthcare utilisation patterns and covariates. For illustration purposes, we focus on the association between membership to the low utilisation cluster and regular lipid testing. The bootstrap procedure produces an AME matrix of dimension 206 × 1000, where the number of rows corresponds to the number of individuals assigned to the low healthcare utilisation cluster in the original analysis. Values are missing in this matrix when a given individual was not sampled in a given bootstrap. Figure 5 presents the empirical distribution of these AMEs and Figure S4 shows their corresponding SEs.^2^ Considering the high estimated cluster-wise stability, we expect most individuals in the reference cluster to have the same estimated AME in a bootstrap, although different values can occur when the original typology is not recaptured or when individuals are assigned to another type.

We applied the multilevel model presented in Eqs. (1) and (2) to pool the results from the 1′000 bootstraps and produce the estimates shown in Table 2. The overall intercept equals 0.233 and represents the expected change in cluster membership probability based on the typology replications for patients who complied with lipid screening recommendations, for an average bootstrap sample and an average patient in the original low healthcare utilisation cluster. It is also called the pooled AME for simplicity. The small SE value indicates as expected that there are enough bootstrap replications, and that despite the results’ randomness caused by the bootstrap sampling, variation in the pooled AME is limited.

**Table 2:** Results from the multilevel model fitted on the AMEs obtained from a 1′000 bootstrap procedure for the association between regular lipid screening and low healthcare utilisation, with the original analysis for comparison

***Original analysis***	*AME*	*0.271*
	*SE*	*0.105*
**Overall intercept fixed effect**	Estimate	0.233
	SE	0.005
**Patient random effect**	SD	0.03
**Bootstrap random effect**	SD	0.127

The standard deviation (SD) of the bootstrap random effect equals 0.127 (Table 2) and is of particular relevance for our analysis. If a new sample is drawn from the same underlying distribution, and a new partition constructed from this sample, the 95% PI for the expected change in cluster membership probability for regular lipid testing based on this new partition *for individuals assigned to the low healthcare utilisation cluster originally* is [−0.016, 0.482] following Eq. (3). This should be compared to the regression estimates in the original analysis: [0.066, 0.476]. The pooled AME is based on a large variety of partitions, which implies different cluster membership variables, reflecting the impact that the sampling uncertainty has on the regression results. Thus, the method uses sampling variation to evaluate the robustness of the original association. While it is expected that both values are not identical, and they are consistent with the intervals here, failing to account for the sampling uncertainty meant that we overestimated the evidence behind the relationship between regular lipid testing and the low healthcare utilisation pattern. Indeed, unlike the original result, the interval obtained using RARCAT now includes the null effect. This finding is corroborated with a training/testing approach in the Supplementary Material.

In some cases, the pooled AME can be smaller in magnitude than the original effect, as indicated when fitting the model on patients belonging to the early deaths cluster in the original analysis (Table 3). Here, the pooled AME is larger than the original estimate, although not discernible from the null effect. Moreover, while there is a substantial amount of variation across bootstraps when considering the association with regular lipid testing, this is reduced when considering an association that has a similar effect size, i.e., with being 75 years old or above (Table 3). In this case, the 95% PI does not contain the null effect, indicating evidence of an effect of age on the probability of belonging to the low healthcare utilisation trajectory group, even after accounting for sampling uncertainty.

**Table 3:** Further results from the multilevel models based on a 1’000 bootstrap procedure, with original values for comparison

Early deaths as reference cluster		
***Original analysis***	*AME*	*-0.049*
	*SE*	*0.061*
**Overall intercept fixed effect**	Estimate	-0.056
	SE	0.002
**Patient random effect**	SD	0.005
**Bootstrap random effect**	SD	0.071
Association with being 75 years old or above		
***Original analysis***	*AME*	*-0.268*
	*SE*	*0.079*
**Overall intercept fixed effect**	Estimate	-0.24
	SE	0.003
**Patient random effect**	SD	0.022
**Bootstrap random effect**	SD	0.089

All the pooled AMEs estimated from a 1′000 bootstrap procedure and their associated 95% PIs are presented in Table S5. While intervals for the associations between low healthcare utilisation and regular lipid testing or age are larger compared to the intervals in Table S3, this is not true for every relationship. For instance, the 95% PI for the association between high healthcare utilisation and diabetes complications at baseline (barely) excludes the null effect, which was not the case for its 95% confidence interval (CI) counterpart from the original analysis. Thus, some relationships seem more immune to sampling uncertainty than others. The association between high healthcare utilisation and regular lipid testing is an example of an unstable association, as it is largely reduced in the robustness assessment.

In summary, while the RARCAT estimates are generally more conservative than the ones from the original “naïve” analysis, the change ultimately depends on which observations are influential in estimating the association of interest, and if those observations are likely to move between clusters based on the sample at hand. In our illustrative application, the three regression results indicating strong evidence of an association (*p* < 0.01; Table S3) all pass the robustness assessment (95% PI; Table S5), but only the relationship between comorbidities and low healthcare utilisation stays virtually unchanged. Out of the five indicating weak evidence of an association (0.01 < *p* < 0.05), only two are lightly impacted and pass the robustness assessment.

**Fig. 6:**
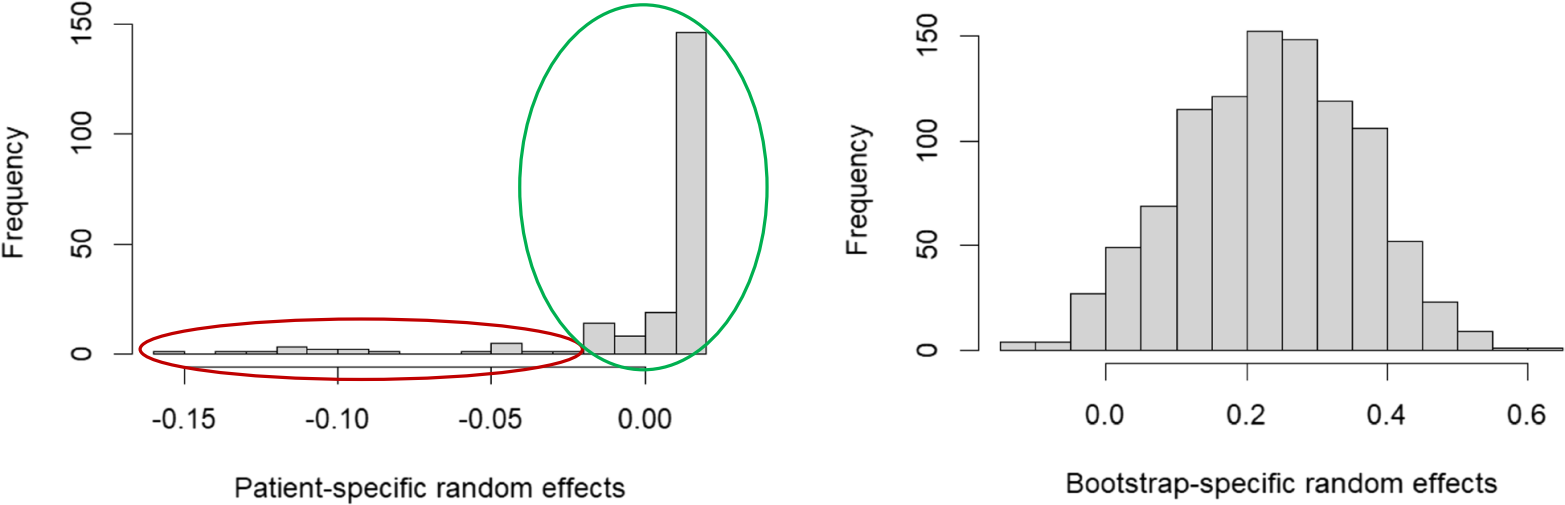
Fitted random effect values from the multilevel model. On the left is the histogram for the 206 patient random effects and on the right, the histogram for the 1′000 bootstrap random effects. The coefficients highlighted in red correspond to “outlier trajectories” and the ones highlighted in green, to “central trajectories”

**Fig. 7:**
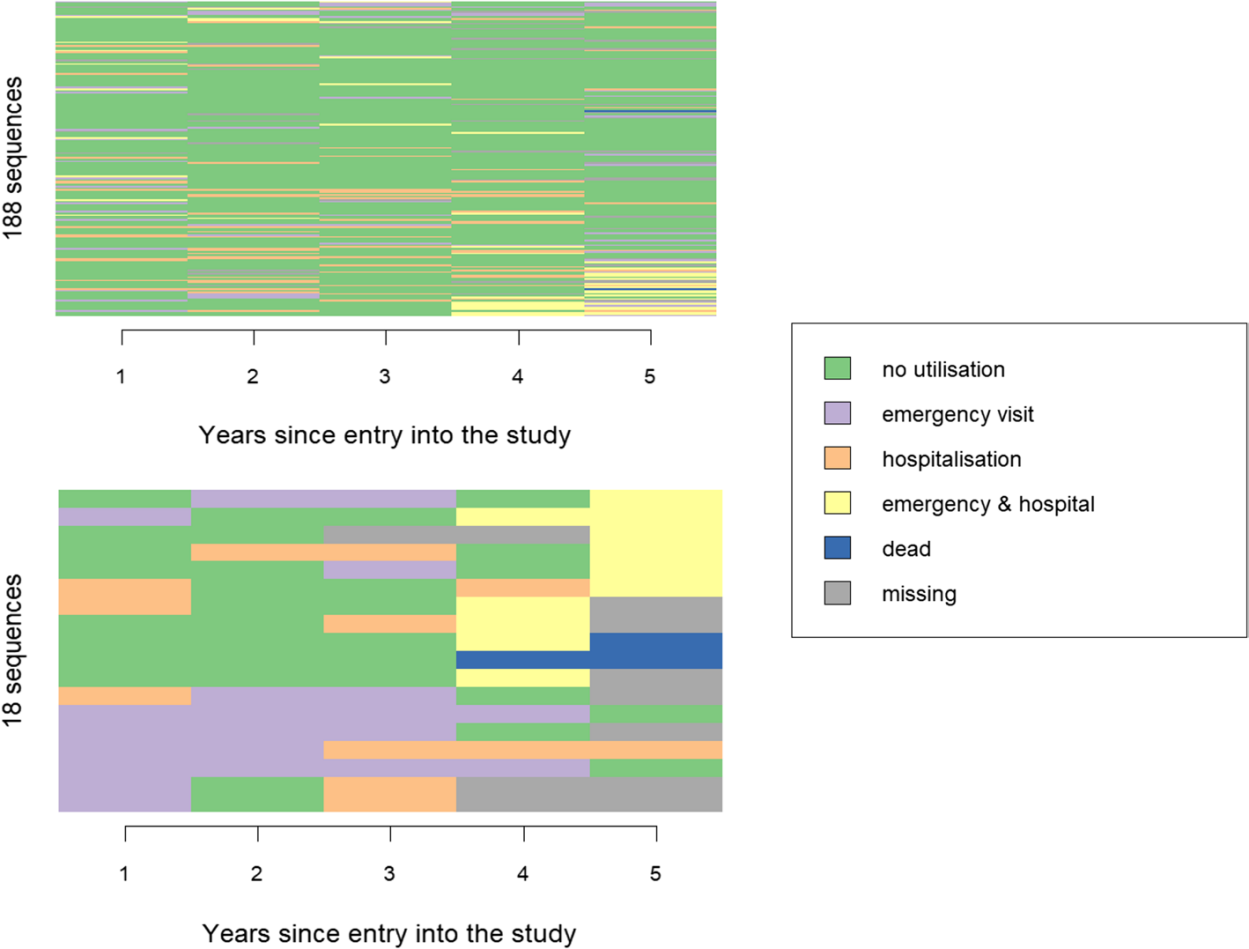
Trajectories from the low healthcare utilisation cluster (*n* = 206). At the top are patients with fitted random effect values close to zero (ordered by magnitude). At the bottom are patients with fitted random effect values far from the average. They indeed appear to be in-between this type and the high healthcare utilisation cluster

Finally, the estimated SD of the individual/patient random effects equals 0.03 (Table 2), which is smaller than the bootstrap random effects one. The fitted values for these random effects, which represent the deviations from the overall intercept, are shown in Fig. 6. Trajectories with a large deviation from the central effect are highlighted in red and interpreted as outlier trajectories in the cluster. In our case, while trajectories corresponding to fitted random effect values below 0.03 (or one SD away from the mean) are mostly characterized by no healthcare utilisation, the ones with fitted values above 0.03 seem to be a mixture of types and could have been assigned to another cluster (Fig. 7). Thus, RARCAT furthermore enables the identification of borderline or atypical cases that may impact the robustness of the association between clusters and covariates. A complementary method using jackknife-after-bootstrap diagnostics to identify individuals exercising a particular influence on the estimated association is presented in the Supplementary Material.

### Alternative specifications

We revisit as alternative specifications of the method three assumptions made when assessing the robustness of the association of interest. First, the patient-specific deviations from the overall intercept were modelled as random effects to express that each individual can have a different underlying true effect. The estimated distribution of these random effects shows many values close to the zero mean and a few diverging values (Fig. 6). As already discussed, another option is to model the individual variations as fixed effects, where no assumption is made on their distribution. Table 4 presents the impact of this change on the multilevel regression, which was estimated on the same set of AMEs as before. Compared to the results in Table 2, the main estimate, i.e., the pooled AME, is slightly increased. However, the 95% PI for a new bootstrap sample still contains the null effect.

**Table 4:** Results from three multilevel models based on two 1′000 bootstrap procedures for alternative specifications of the method, with original values for comparison

***Original analysis***	*AME*	*0.271*
	*SE*	*0.105*
Patient-specific fixed effects		
** Overall intercept fixed effect**	Estimate	0.245
	SE	0.005
** Bootstrap random effect**	SD	0.127
Inverse hyperbolic tangent transformation		
** Overall intercept fixed effect**	Estimate	0.238
	SE	0.004
** Patient random effect**	SD	0.031
** Bootstrap random effect**	SD	0.136
Varying number of clusters		
** Overall intercept fixed effect**	Estimate	0.199
	SE	0.005
** Patient random effect**	SD	0.023
** Bootstrap random effect**	SD	0.134

Second, the original analysis fitted a linear multilevel model on the AMEs from the bootstrap procedure. However, these are bounded so the model might not be proper in certain extreme cases. An inverse hyperbolic tangent transformation applied on the AMEs circumvent this issue. Table 4 shows the quantities estimated with such a model. As expected, they are close to the ones from Table 2.

Finally, as already mentioned, RARCAT also allows to vary the optimal number of clusters obtained in each bootstrap replication. By doing so, we account for the sampling uncertainty’s impact on the estimation of the number of groups *K* from the data. The corresponding results are shown in Table 4. Logically, it introduces more variability in the typologies across bootstraps. While the three clusters solution was selected for most bootstrap samples, the two and four clusters solution were also chosen in some bootstrap replications based on the CHI. The rest of the procedure stays identical.

Results from the first two alternative specifications (patient-specific fixed effects and inverse hyperbolic tangent transformation) are very close to the main analysis ones, so apart from specific situations such as peculiar distributions of the AMEs in the bootstrap output or extreme associations, we recommend going with the simpler and more natural version, i.e., the main one. However, while the number of clusters was for the most part assumed to be fixed here for the sake of simplicity, it is more suitable to estimate it in each bootstrap if it was part of the modelling process, so we recommend considering the corresponding specification as a generalisation of RARCAT.

## Discussion

In this article, we proposed a new set of methods labelled as RARCAT to assess the robustness of typology-based inference. The novel procedure was illustrated by investigating the relationship between healthcare utilisation patterns and covariates of interest for diabetic patients. For illustration purposes, we examined primarily the effect of regular lipid testing on membership to the low healthcare utilisation cluster, controlling for known confounders.

At the core of the proposal is an attempt to measure the impact of sampling error on a standard SA framework. To achieve this goal, a bootstrap procedure is implemented, which allows to reconstruct the clustering on an ensemble of datasets resampled from the same approximating distribution. The new partitions are used to assess the original typology by evaluating cluster-wise stability through average Jaccard similarities, as it was done in previous works (e.g., [26, 58]).

We propose to go one step further by estimating regression models for the association between the clustering and covariates of interest for each bootstrap sample. Then, based on the meta-analysis concepts, the regression results are pooled with multilevel modelling. Crucially, as the quantities estimated in the bootstrap procedure are not directly comparable across bootstraps, they are considered at the individual level. The appraisal is then operated with reference to the original typology. It is therefore cluster-wise by design.

The output of the multilevel model sheds light on the original analysis in two key ways. First and foremost, the average effects and their 95% PIs based on the bootstrap random effects constitute new estimates for an association of interest, which account for the sampling uncertainty. Second, the individual random effects inform on the central and outlier trajectories in a cluster. Identifying trajectories that are not properly assigned is valuable in many situations.

### Case study assessment

The illustrative application led to various findings after applying RARCAT. In the main analysis, while the cluster-wise stability was generally high, the 95% PI for the association between regular lipid testing and low healthcare utilisation during follow-up contained the null effect, which is in opposition with the original estimates. By contrast, the 95% PI for the association between being 75 years old or above and membership to the low healthcare utilisation cluster did not contain the null effect, although it was also larger than its 95% CI counterpart. Other associations such as the one between diabetes complications and healthcare utilisation patterns did not suffer from the same reduction in effect sizes. This is an indication that, besides the impact of sampling uncertainty on the clustering itself, it also has a differential impact on subsequent inference depending on the association of interest. Indeed, some relationships appear more robust to sampling variation than others.

Coming back to the case study, evidence of regular lipid testing’s positive effect on avoiding future adverse healthcare events did not withstand the robustness assessment. We conclude that the original effect was magnified due to inadequate handling of sampling error in the standard SA framework. This led to an overconfidence in the strength of the association. The quantities estimated thanks to RARCAT are consistent with a previous study on the topic, which found no association between lipid control and diabetes-related hospitalisations [59].

However, several limitations may influence the findings from our illustrative application. First, the study period was narrow, so the recommended care processes were only considered for the first two years. Second, we did not possess precise information on the reasons for emergency visits, hospitalisations nor deaths. Third, all data were self-reported, which implies a risk of recall bias. Fourth, even if the cohort was nearly representative originally [37], attrition and repeated missing observations meant that some individuals were not included in the analysis. For all these reasons, the results should not be understood beyond their usefulness as a case study.

### Methodological implications

Any study that identifies a typology and uses it for subsequent inference can be enhanced by our methodological proposal. Thus, it can be applied in many situations, as this framework is arguably the most prevalent and fundamental in SA. In particular, the methods are agnostic on the dissimilarity measure, clustering algorithm or cluster quality index, so any common SA procedure can be used. However, the results still depend on the choice of clustering, and as noted by Hennig [26], an inflexible clustering algorithm that would yield artificially stable results should be avoided. Moreover, it can sometimes make sense to study the relationship between trajectories and covariates without going through the clustering step (see for instance [16]). However, whenever a sensible typology is the starting point, the findings’ reliability can be assessed with RARCAT. Handling fuzzy clustering or using the typology as explanatory variable would necessitate extensions of the method, which calls for further research.

The methodological developments led to the derivation of new quantities that are more robust to sampling uncertainty. Sampling uncertainty can impact an analysis at different levels. First, the sample size is important as larger samples will reduce the sampling error. Second, the macro-structure of the data matters as the clustering is more likely to be reproduced over new samples from the same underlying population if there are clear and distinct groups, i.e., homogeneous and well-separated. In the opposite case, the cluster centres may vary greatly between samples. At the micro-level too, individual sequences that do not belong to any homogeneous group or in-between several risk being classified differently in each sample. These considerations highlight the sample dependency of the clustering. Moreover, inside the same clustering, this dependency does not affect every cluster identically.

All the above influence subsequent inference and its stability. Furthermore, as we saw with our illustrative application, certain relationships are more at risk of being impacted by sampling variation than others. This means that removing and/or adding a few observations in a cluster can change the regression results in different ways depending on the covariate of interest. Being able to detect associations that are more, or less, robust to sampling uncertainty is a strength of our proposal.

The bootstrap procedure is justified because the true data generating process is unknown. Thus, the sample on hand is the best approximation of the underlying population. This has implications on the interpretation of the PI constructed from the multilevel model. A bootstrap sample can be envisioned as a potential new sample to validate the analysis on. The PI gives our best estimate of the interval in which the association of interest will fall, considering that the typology may change in the new sample. Thus, it is of great significance for assessing the reproducibility of the analysis.

The distributional properties of the bootstrap procedure output, i.e., the AMEs for each combination of bootstrap and individual as presented in Fig. 5, are unclear at this point. It can potentially impact the adequacy of fitting a linear mixed-effects model to these results. We have already seen in Fig. 6 that the assumption of normality of the individual-specific random effects is infringed, as evident from the left-skewness. This is a limitation of RARCAT. However, as pointed out by Schielzeth et al. [53], linear mixed-effects models are robust to violations of distributional assumptions in many cases akin to ours.

It is possible to situate our contribution inside a wider cluster analysis validation framework [28]. In this context, the validation data are the bootstrap samples, the properties to be validated are associations of the clusters with external variables and the validation is method-based, even though the terminology can seem a bit confusing. Studies of this sort typically validate results on a separate dataset containing new observations [28]. To the best of our knowledge, we are the first to use bootstrap samples to assess external associations in the context of clustering. Taking inspiration from meta-analysis was key to achieve this aim.

Judging validation success is non-trivial [28]. In our case, an association of interest is considered robust to sampling uncertainty if the effect estimated with RARCAT provides evidence for the claim about the effect in the original study. This occurs when the PI based on the bootstrap random effects is coherent with the original CI. Our approach is in line with recommendations from the research on replication studies [60].

While our proposed method is a direct answer to a clear shortcoming of classical SA, its efficacy under different parameters still needs to be evaluated, including different sample sizes, clustering structures or levels of association. This article focuses on the theoretical support for the approach taken but its practical merit could be further assessed. A complementary study based on out-of-sample predictions is presented in the Supplementary Material. It confirms the instability of the association found with the methodological framework we introduced, although the information it provides is somewhat different. Nonetheless, future research involving RARCAT with simulations or various case studies would strengthen our methodological development and deepen our understanding of its capabilities.

### Conclusion

While clustering is a statistical technique that has many applications and is still gaining in popularity, it is also a challenging task with several potential pitfalls. Adding an inferential element when investigating relationships between clusters and covariates brings further challenges to it. Common SA studies must navigate these different risks. In this article, we have reviewed previous works proposing innovative ways to handle the data reduction risk involved when building a typology and using it in subsequent inference. Acknowledging the dearth of literature on the impact of sampling error in standard SA, we propose a RARCAT method to assess the robustness of regression results based on SA typologies. It is complementary to other methods that have been introduced in recent years to improve the reliability of SA’ every steps. We hope that many SA researchers will find our methodology useful and recommend its adoption wherever it is applicable. We also hope that the procedure will find an echo beyond the world of SA and influence future works aiming to use the bootstrap to validate results in complex statistical settings.

## Supplementary Information

Supplementary Material 1.

## Data Availability

The data and code that support this study are available from the corresponding author, Leonard Roth, upon reasonable request. Contact: leonard.roth@unisante.ch.

## Abbreviations

AME: Average marginal effect
CHI: Calinski-Harabasz index
CI: Confidence interval
PI: Prediction interval
RARCAT: Robustness Assessment of Regressions using Cluster Analysis Typologies
SA: Sequence analysis
SD: Standard deviation
SE: Standard error

## Footnotes

1 For instance, rather than looking at the association between regular lipid testing and general cluster membership, we centre our analysis on a specific cluster and investigate how it is characterised by previous lipid testing compliance.

2 Each bootstrap replication of the four analysis steps (resampling, clustering, regression modelling, retrieving the AMEs) takes just below two seconds to run here, so that the whole 1′000 bootstrap procedure has a total computation time of about 30 min on a laptop and without parallelisation. Larger sample sizes will imply larger computation times, although different strategies can be employed to increase the efficiency of the clustering step (see e.g. [61]).
