# Supplementary material for: Robustness assessment of regressions using cluster analysis typologies: a bootstrap procedure with application in state sequence analysis
Source: BMC Med Res Methodol. 2024 Dec 18;24:303. doi: 10.1186/s12874-024-02435-8 (PMC11654397; doi:10.1186/s12874-024-02435-8)
Supplement: Supplementary file 1 — Supplementary Material 1. [file 12874_2024_2435_MOESM1_ESM.pdf]

Supplementary Material

Robustness Assessment of Regressions using Cluster Analysis Typologies:

A Bootstrap Procedure with Application in State Sequence Analysis

Page 2: Supplementary Table S1

Page 3: Supplementary Figure S2

Page 4: Supplementary Table S3

Page 5: Supplementary Figure S4

Page 6: Supplementary Table S5

Page 7: Out-of-sample evaluation of the association

Page 9: Bootstrap diagnostics - outliers

Variable	Type	Values
Age	Categorical	<65 years old; 65–74 years old; >=75 years old
Household income	Categorical	low; lower-middle; upper-middle; high
Diabetes treatment	Categorical	oral antidiabetic medication (OAD); insulin; both
Diabetes-related complications	Integer	*N* among ischemic heart diseases, stroke, retinopathy, chronic kidney disease (CKD) without dialysis, CKD with dialysis or kidney transplant, neuropathy, foot ulcer, lower limb amputation, severe hypo- or hyperglycaemia
Comorbidities	Integer	*N* among heart disease, chronic lung disease, osteoporosis, osteoarthritis or arthritis, cancer or malignancy or lymphoma, gastric or duodenal ulcer, depression, Parkinson disease, hypertension, hyperlipidaemia

Supplementary Table S1: Covariates for the association between compliance to recommended care processes and healthcare utilisation patterns.

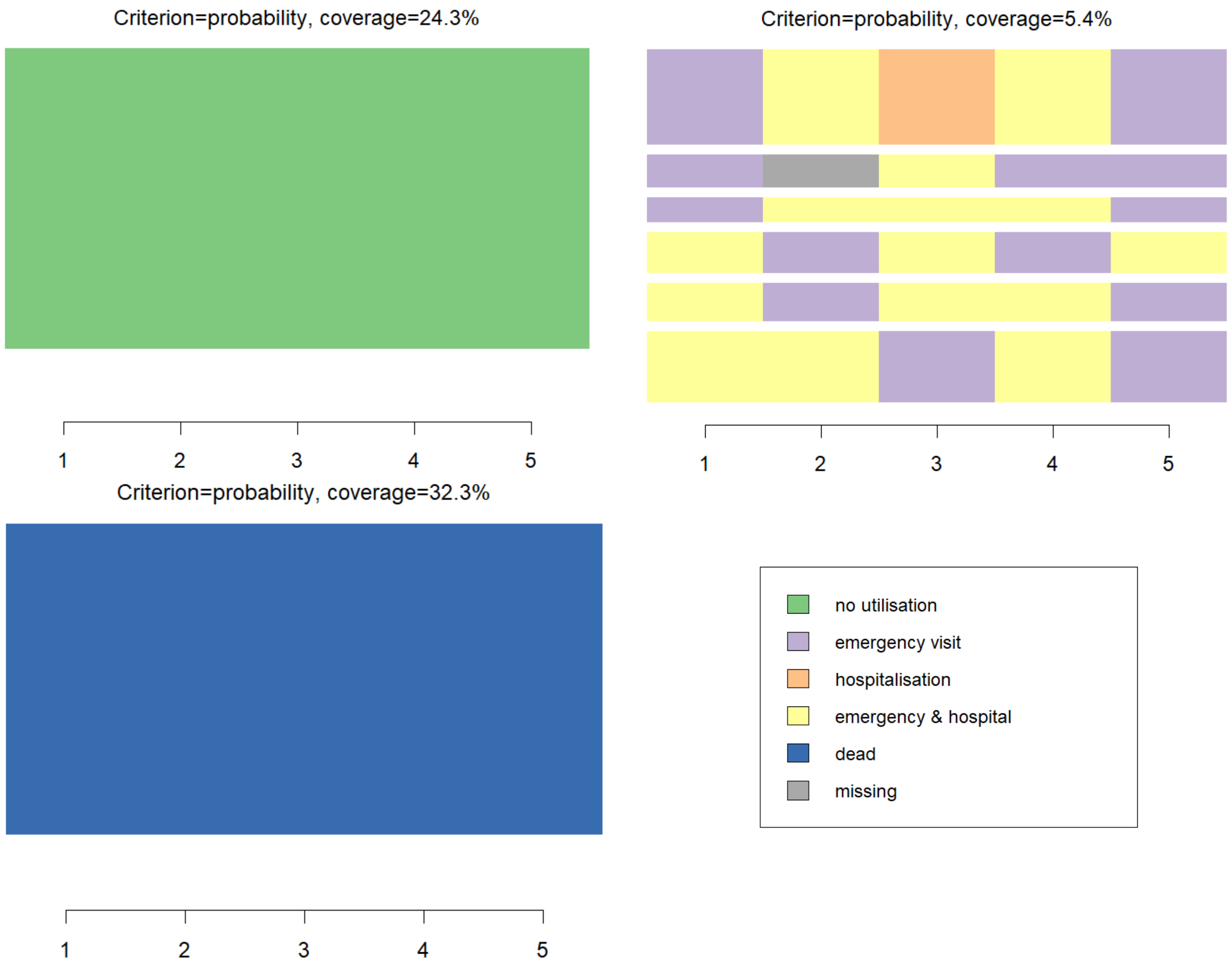

Supplementary Figure S2: Most representative sequences (in terms of silhouette value) with at least 5% coverage for the three clusters identified. X-axis represents the five years of follow-up (2013 - 2017). Top left is the low (intermittent) healthcare utilisation cluster (n = 206). Top right is the high (intensive) healthcare utilisation cluster (n = 111). Bottom left is the early deaths cluster (n = 31).

		Low healthcare utilisation	Intensive healthcare utilisation	Early deaths
**Lipid testing**	yes (vs. no)	0.271** [0.066, 0.476]	-0.209* [-0.424, 0.007]	-0.049 [-0.169, 0.071]
**Age (years)**	65-74 (vs. <65)	-0.075 [-0.185, 0.034]	0.009 [-0.098, 0.116]	0.074** [0.016, 0.131]
	>=75 (vs. <65)	-0.268*** [-0.423, -0.112]	0.044 [-0.112, 0.199]	0.225*** [0.1, 0.35]
**Household income**	lower-middle (vs. low)	0.029 [-0.124, 0.182]	0.084 [-0.063, 0.231]	-0.093** [-0.184, -0.003]
	upper-middle (vs. low)	0.046 [-0.108, 0.199]	0.029 [-0.115, 0.173]	-0.063 [-0.161, 0.036]
	high (vs. low)	0.073 [-0.097, 0.242]	0.037 [-0.126, 0.2]	-0.104** [-0.201, -0.007]
**Diabetes treatment**	insulin (vs. OAD)	-0.04 [-0.178, 0.098]	-0.031 [-0.16, 0.097]	0.058 [-0.025, 0.142]
	both (vs. OAD only)	-0.103* [-0.223, 0.018]	0.068 [-0.054, 0.189]	0.03 [-0.041, 0.101]
**Diabetes complications**	N	-0.061** [-0.114, -0.007]	0.046* [-0.006, 0.099]	0.022 [-0.007, 0.051]
**Comorbidities**	N	-0.053*** [-0.089, -0.017]	0.035* [-0.001, 0.071]	0.015 [-0.004, 0.035]

Supplementary Table S3: Results (n = 342) from the three logistic regression models with membership to a specific cluster as the dependent variable. Coefficients are average marginal effects (AMEs) representing the expected change in probability of belonging to the trajectory group for a change in the level of a variable, after adjustment for the other covariates. Confidence intervals are at a 95% level. *p < 0.1; **p < 0.05; ***p < 0.01.

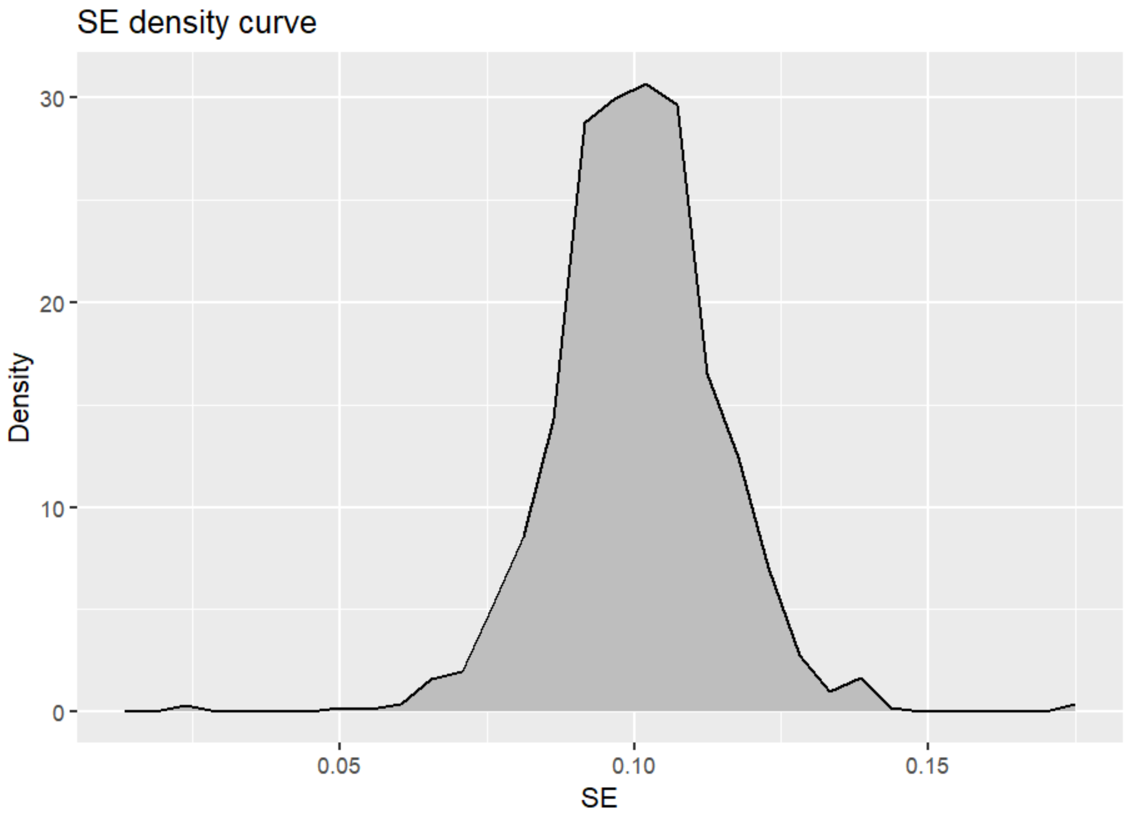

Supplementary Figure S4: Density plot for 129’829 saved standard errors (SEs) from a 1’000 bootstraps procedure. Each SE corresponds to an estimated AME for the association of interest from Figure 5.

		Low healthcare utilisation	Intensive healthcare utilisation	Early deaths
**Lipid testing**	yes (vs. no)	0.233 [-0.016, 0.482]	-0.145 [-0.41, 0.12]	-0.056 [-0.195, 0.083]
**Age (years)**	65-74 (vs. <65)	-0.051 [-0.171, 0.069]	-0.018 [-0.128, 0.092]	**0.075 [0.012, 0.138]**
	>=75 (vs. <65)	**-0.24 [-0.414, -0.066]**	0.016 [-0.164, 0.196]	**0.21 [0.075, 0.345]**
**Household income**	lower-middle (vs. low)	0.034 [-0.121, 0.189]	0.069 [-0.068, 0.206]	-0.084 [-0.18, 0.012]
	upper-middle (vs. low)	0.048 [-0.115, 0.211]	0.025 [-0.114, 0.164]	-0.061 [-0.165, 0.043]
	high (vs. low)	0.08 [-0.092, 0.252]	0.03 [-0.119, 0.179]	-0.099 [-0.199, 0.001]
**Diabetes treatment**	insulin (vs. OAD)	-0.036 [-0.161, 0.089]	-0.036 [-0.154, 0.082]	0.061 [-0.025, 0.147]
	both (vs. OAD only)	-0.104 [-0.233, 0.025]	0.063 [-0.072, 0.198]	0.035 [-0.045, 0.115]
**Diabetes complications**	N	**-0.07 [-0.131, -0.009]**	**0.048 [0.001, 0.095]**	0.02 [-0.009, 0.049]
**Comorbidities**	N	**-0.053 [-0.092, -0.014]**	0.033 [-0.012, 0.078]	0.014 [-0.006, 0.034]

Supplementary Table S5: Reproduction of Table S2 based on the robustness assessment with a 1’000 bootstrap procedure. The coefficients are pooled AMEs and the 95% prediction intervals, which give a range for the predicted parameter value for a new sample from the same underlying distribution, are approximated based on the standard deviation of the bootstrap random effects. Prediction intervals that do not contain the null effect are indicated in bold.

Out-of-sample evaluation of the association

Here, we illustrate the shortcomings of the original analysis through a training/testing approach. The general idea is to test how well an association of interest estimated on training data can be translated out-of-sample. To do this, we evaluate its prediction accuracy on testing data by calculating the Area Under Curve (AUC) of the Response Operating Characteristic (ROC) curve, a common measure in this context[1]. Specifically, the procedure works as follows:

1. Split the data in two, with two thirds (approximately 67%) used for training and the rest (approximately 33%) used for testing.
2. Apply the clustering algorithm used in the original analysis (Partition Around Medoids with Optimal Matching) on the train set. Assign each observation in the test set to a cluster based on its shortest distance with the cluster centres.
3. Fit the regression model on the train set for an association of interest between the clustering and a covariate.
4. Predict cluster assignment for the test set based on the fitted regression model and calculate the ROC-AUC by comparing the predicted values with the actual ones obtained in step 2.
5. Compute the p-value associated with this ROC-AUC with a Mann-Whitney U test. This answers the question: is there evidence that the covariate of interest predicts the clustering on the test set?

To get a better understanding of what this does, let us first apply this procedure without data splitting. The clustering is the same as in the original analysis and we consider the association between regular lipid screening and membership to the low healthcare utilisation cluster. Now, we can predict cluster assignment based on the fitted coefficients for this association, which gives a relatively high probability to belong to the low healthcare utilisation cluster for the individuals who reported regular lipid screening (the large majority), and a relatively low probability for the rest. We can compare those predictions with the actual cluster assignments and compute the ROC-AUC. The exact value of this performance measure is not relevant here, but the corresponding p-value is < 0.01, which could be expected considering the association found between these two variables in the original analysis. Note that we find a similar p-value (< 0.01) for the predictive accuracy of age (three categories).

These results are not particularly interesting *per se* as everything was done in sample. There is randomness involved in the step-by-step training/testing procedure presented above, due to the random data splitting, so we repeat it a large number of times (n = 1’000). The output is a vector of 1’000 p-values indicating for each repetition whether there is evidence that regular lipid screening predicts cluster membership out-of-sample in our data. A histogram of these p-values is shown in Supplementary Figure S6 below. The median p-value is 0.07. This tells us that within these 1’000 iterations, the value 0.5 (fully random prediction) was contained in more than half of the 95% confidence intervals for the ROC-AUC. The same procedure applied with age as covariate gives the p-values in Figure S7. This time, the median p-value is 0.03.

[1] Fawcett T. An Introduction to ROC Analysis. Pattern Recognition Letters. 2006;27:861-874.

To conclude, it is of course expected that the prediction accuracy decreases out-of-sample compared to in-sample. Nevertheless, if we only base ourselves on the original association, we deduce that there is clear evidence of a relationship between regular lipid screening and subsequent healthcare utilisation. However, this underestimates the impact of the uncertainty involved in the clustering process, which is again manifest here. Thus, both the main RARCAT procedure and the one outlined here lead to the same findings. RARCAT is a more complete framework though, allowing for a full adjusted regression and leading to the estimation of new quantities for the associations of interest.

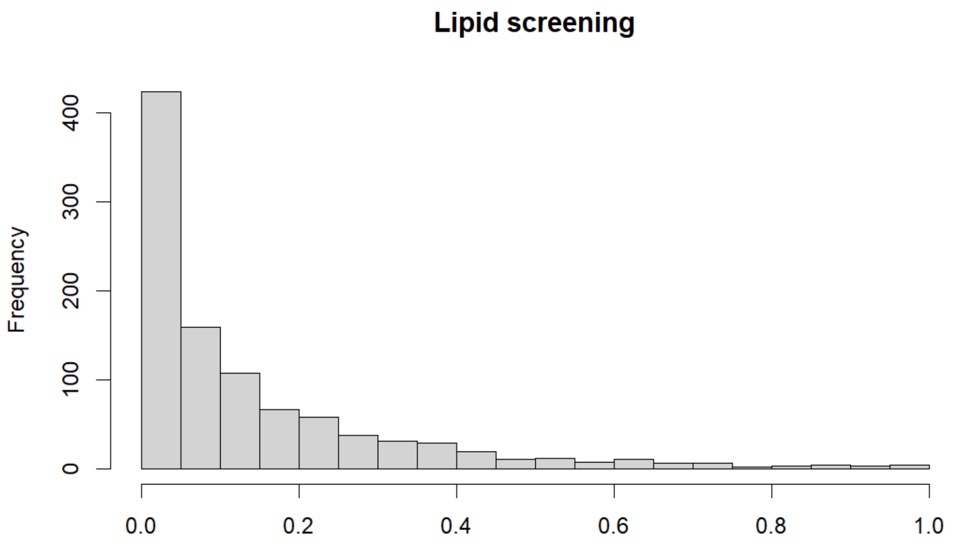

Supplementary Figure S6: Histogram of the p-values corresponding to the 1'000 AUC ROC curves for the out-of-sample evaluation of the association with regular lipid screening.

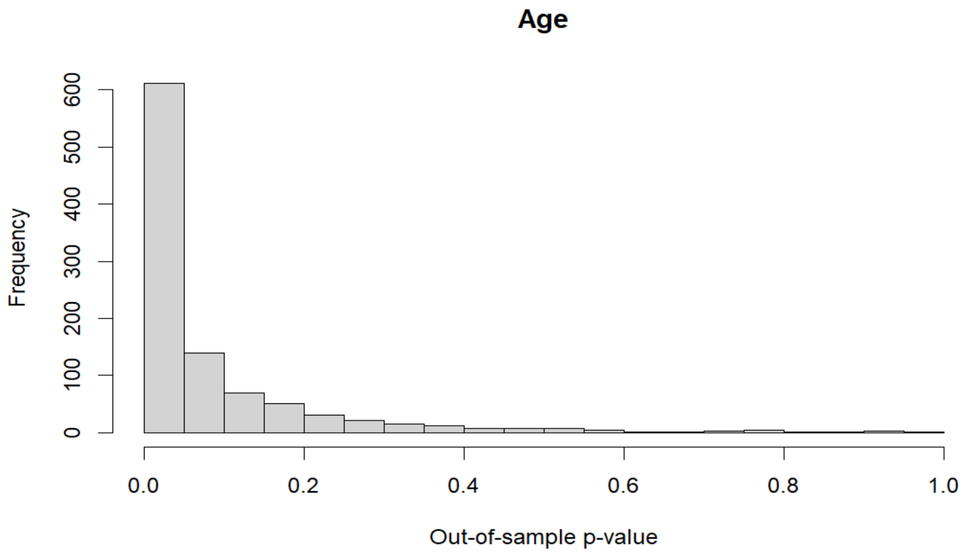

Supplementary Figure S7: Histogram of the p-values corresponding to the 1'000 AUC ROC curves for the out-of-sample evaluation of the association with age.

Bootstrap diagnostics - outliers

To evaluate the impact of outliers on the RARCAT procedure, we take inspiration from the bootstrap diagnostics discussed in Canty AG et al. (2006)[2]. In particular, the *jackknife-after-bootstrap* approach proposes to redo the analysis by omitting one individual, thus enabling to compare the results with and without that individual. Every individual appears in approximately 63.2% of the bootstraps (theoretical value, see Methods in the main document), so it leaves approximately 36.8% of the bootstraps each time for the diagnostics.

In our case, the bootstraps were used to compute an AME matrix with an entry for each individual and each bootstrap, and these quantities were in turn used to estimate a multilevel model to derive the pooled AME and its 95% PI, among others. The same process can be applied for every individual on the subset of the AME matrix where this individual does not appear (i.e., considering only the bootstraps where she/he was not sampled). Thus, a "jackknife pooled AME" as well as the other quantities from the multilevel model are estimated for each individual.

Our situation is quite specific, with the bootstraps being first used for clustering, then for regression based on the typology, and then combined to obtain a summary measure. Nevertheless, we can compute a standardized jackknife influence value for each individual, which corresponds to the standardized distance between her/his estimated jackknife pooled AME and the average one. These values are shown in Supplementary Figure S8 (next page), where the x-axis represents all individuals ordered by their jackknife value. These diagnostics are only mildly informative, so to get a better understanding of the impact of the outliers (the ones with large jackknife values), we reproduce the full analysis, i.e., look at the jackknife pooled AMEs with their corresponding 95% jackknife PIs. Those are shown (with the same ordering of the individuals) in Figure S9, where the central line represents the original pooled AME (i.e., with all individuals and bootstraps considered) and the outside dotted lines, the corresponding limits in the original 95% PI. We see that most jackknife intervals are quite close, especially considering that they were estimated on a significantly lower number of bootstrap replicates (in practice, between 331 and 410 here), and that the individuals with larger jackknife influence values are also discernible in this case. In fact, these individuals have a healthcare utilisation trajectory close to the centre of the cluster (so with mostly no utilisation), but did not report regular lipid screening, unlike most individuals in this cluster (see Table 1). Thus, ignoring them in the analysis pushes the results towards a stronger association between regular lipid screening and low healthcare utilisation trajectories. Note that the "outliers" identified here are not the same as the outliers in terms of fitted random effect values in Figure 6 in the main document, as they affect the analysis on another level (individuals with divergent lipid screening information versus trajectories in-between clusters).

[2] Canty AJ, Davison AC, Hinkley DV, Ventura V. Bootstrap diagnostics and remedies. The Canadian Journal of Statistics. 2006;34(1):5-27.

To conclude, while there is evidence here of influential outliers in our analysis, this could be expected considering the relatively small sample size, and it does not shadow the effectiveness of RARCAT. Indeed, this is a feature of the original clustering and associated regression model, and the bootstrap procedure is behaving appropriately in this aspect.

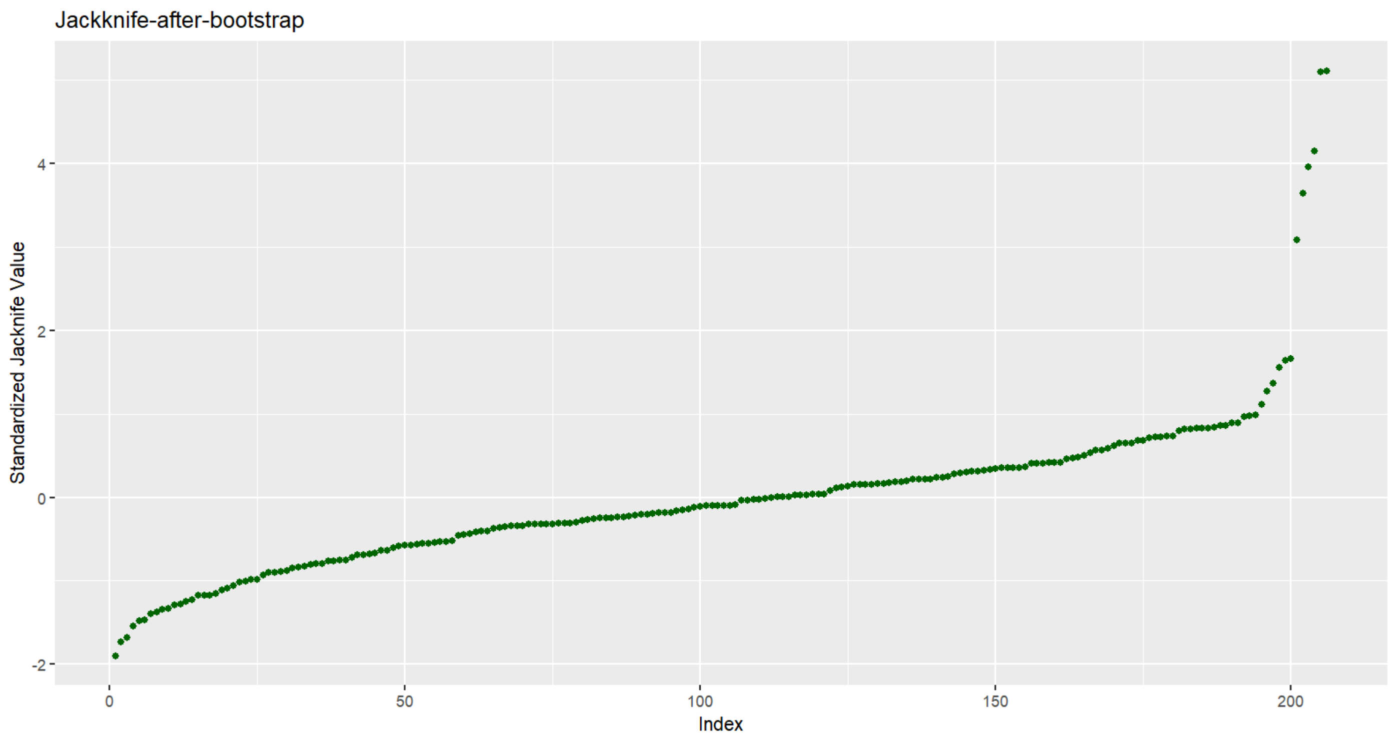

*Supplementary Figure S8: S*tandardized jackknife values for the 206 individuals in the LHU cluster

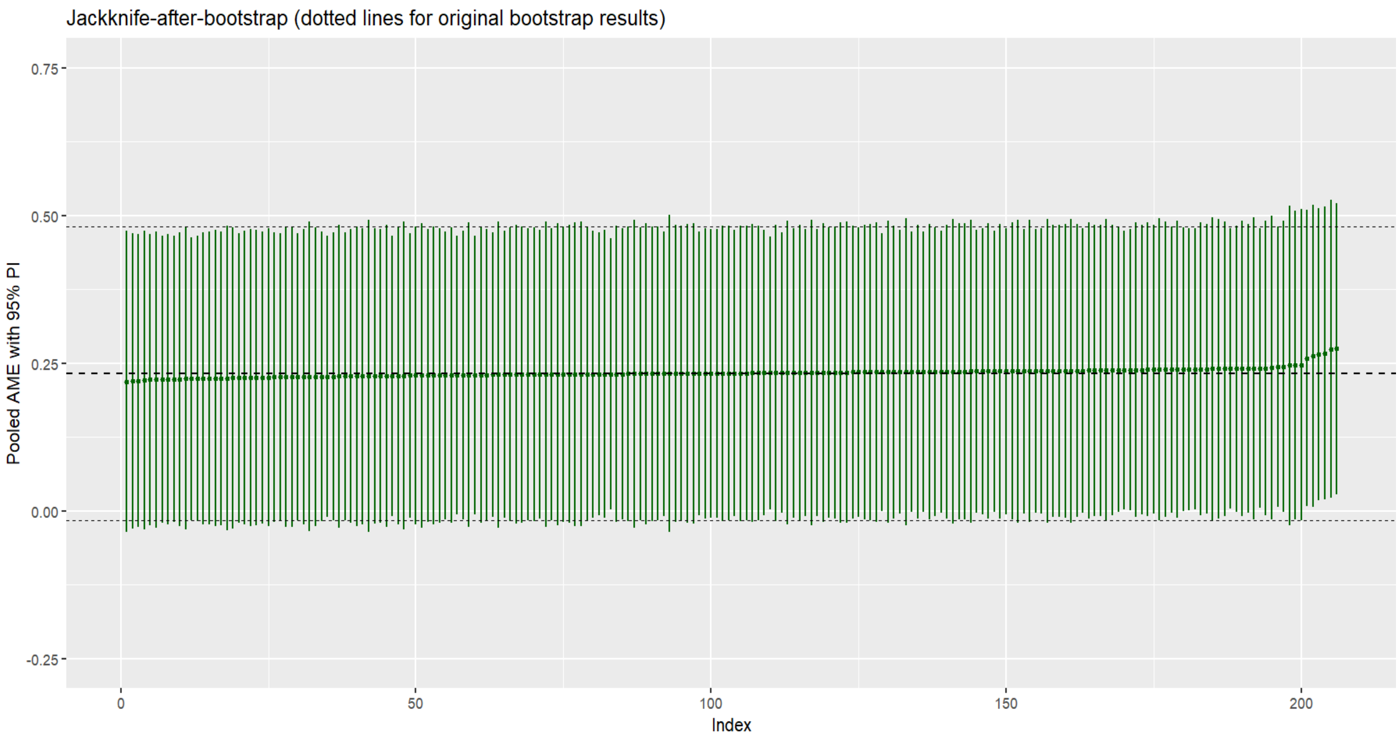

Supplementary Figure S9: Jackknife RARCAT results for the 206 individuals in the LHU cluster
